# Genome-wide mapping of the RNA targets of the *Pseudomonas aeruginosa* riboregulatory protein RsmN

**DOI:** 10.1093/nar/gky324

**Published:** 2018-04-30

**Authors:** Manuel Romero, Hazel Silistre, Laura Lovelock, Victoria J Wright, Kok-Gan Chan, Kar-Wai Hong, Paul Williams, Miguel Cámara, Stephan Heeb

**Affiliations:** 1 School of Life Sciences, Centre for Biomolecular Sciences, University Park, University of Nottingham, Nottingham NG7 2RD, UK; 2 International Genome Centre, Jiangsu University,Zhenjiang, China; 3 Division of Genetics and Molecular Biology, Institute of Biological Sciences, Faculty of Science, University of Malaya, Kuala Lumpur, Malaysia

## Abstract

Pseudomonads typically carry multiple non-identical alleles of the post-transcriptional regulator *rsmA*. In *Pseudomonas aeruginosa*, RsmN is notable in that its structural rearrangement confers distinct and overlapping functions with RsmA. However, little is known about the specificities of RsmN for its target RNAs and overall impact on the biology of this pathogen. We purified and mapped 503 transcripts directly bound by RsmN in *P. aeruginosa*. About 200 of the mRNAs identified encode proteins of demonstrated function including some determining acute and chronic virulence traits. For example, RsmN reduces biofilm development both directly and indirectly via multiple pathways, involving control of Pel exopolysaccharide biosynthesis and c-di-GMP levels. The RsmN targets identified are also shared with RsmA, although deletion of *rsmN* generally results in less pronounced phenotypes than those observed for *ΔrsmA* or *ΔrsmArsmN*_ind_ mutants, probably as a consequence of different binding affinities. Targets newly identified for the Rsm system include the small non-coding RNA CrcZ involved in carbon catabolite repression, for which differential binding of RsmN and RsmA to specific CrcZ regions is demonstrated. The results presented here provide new insights into the intricacy of riboregulatory networks involving multiple but distinct RsmA homologues.

## INTRODUCTION

Environmental fluctuations drive the development of co-ordinated sensory responses to facilitate bacterial survival and growth in challenging conditions. Responding to such challenges is especially important for pathogenic bacteria with respect to evasion of host immune responses, acquisition of limiting nutrients and competition with commensal bacteria. The opportunistic pathogen *Pseudomonas aeruginosa* has multiple sensory systems that allow it to adapt by integrating different sensory inputs (1). For example, two-component systems (TCSs) that employ membrane-integrated sensors that constantly sample environmental signals and activate cytoplasmic transcriptional regulators through phosphotransfer or phosphorelay mechanisms are especially abundant in *P. aeruginosa* (2).

Among the >60 TCSs predicted within the genome of *P. aeruginosa*, the GacS/GacA TCS can channel signals from a range of sensors so acting as an integrating global regulator of multiple pathways. This enables *P. aeruginosa* to adopt different free-living or biofilm associated lifestyles, appropriate for establishing acute or chronic infections. A molecular signal(s) that has yet to be chemically characterized induces autophosphorylation of the sensor kinase GacS and the transfer of a phosphate group to its cognate regulator GacA. This in turn induces the transcription of the small regulatory RNAs (sRNAs), RsmY and RsmZ. These sRNAs sequester the RNA-binding protein RsmA, a key post-transcriptional regulator that impacts either positively or negatively on the translation rates of multiple transcripts in *P. aeruginosa* (∼9% of the gene transcripts, (3)).

RsmA, a homologue of the CsrA family of post-transcriptional regulators originally described in *Escherichia coli* (4), exists as a small compact homodimer (∼14 kDa) and can interact with two RNA motifs, with a strong preference for ANGGA-containing sequences situated within RNA hairpins (5–8). RsmA represses the translation of *P. aeruginosa* genes required for the establishment of chronic biofilm-centered infections including those encoding for type VI secretion systems (T6SS) and exopolysaccharide (EPS) biosynthesis (3,9,10). In addition, RsmA positively impacts on acute infection-related phenotypes including motility and type III secretion systems (T3SS) (11,12). RsmA also controls the intracellular levels of the second messenger cyclic diguanylate (c-di-GMP) (13), which modulates the transition from the planktonic to the biofilm state (14), as well as expression of the cAMP/virulence factor regulator (Vfr) pathway. The latter positively regulates the production of multiple virulence determinants including Type II and III secretion systems (T2SS, T3SS), type IV pili and secreted exoproteases and exotoxins (15).

In contrast to other bacterial genera, Pseudomonads typically retain multiple non-identical copies of *rsmA* in their genomes. The protein structure and RNA specificity among these RsmA homologues within a species enable gene-specific control at the post-transcriptional level and so facilitate rapid survival responses in hostile conditions (16,17). In 2013, a unique *P. aeruginosa* RsmA/CsrA homologue was described and termed RsmN to distinguish it from homologues that more closely resemble the CsrA protein of *E. coli* (18) or RsmF (19). The structure of RsmN incorporates a uniquely inserted α-helix that redirects the polypeptide chain to form a distinctly different protein fold to the domain-swapped dimeric structure of RsmA, even though the overall composition of the protein (five β-strands and an α-helix per monomer) and the RNA-binding pocket are conserved. Moreover, RsmN also binds to GGA-motif-containing hairpins from RsmY and RsmZ *in vitro* with high affinity (18,19).

Despite this, little is known about its RNA-binding affinities and specificities since only a limited subset of RsmA-related targets were used to characterise its RNA-binding activity. Furthermore, a SELEX-derived consensus sequence for RsmN binding is a poor predictor of RsmN targets in the *P. aeruginosa* genome (8). Although SELEX approaches have been effective in finding artificial sequences that avidly bind to the Csr/Rsm proteins, many natural targets have been found to diverge significantly from the consensus sequences so deduced. Here, we identify a genome-wide set of potential RsmN targets by recovering and sequencing the RNAs bound to this protein in *P. aeruginosa*. This approach has enabled us to gain new insights into the biological function of this unique post-transcriptional regulator and its relationship with RsmA.

## MATERIALS AND METHODS

### Bacterial strains, culture conditions and genetic methods

Strains, plasmids and oligonucleotides used in this study are listed in Supplementary Table S1. *E. coli* and *P. aeruginosa* strains were routinely grown at 37°C on lysogeny broth (LB) or LB agar supplemented with antibiotics as required.

For carbon catabolite repression experiments, a basal salts medium (BSM) [30.8 mM K_2_HPO_4_, 19.3 mM KH_2_PO_4_, 15 mM (NH_4_)_2_SO_4_, 1 mM MgCl_2_ and 2 μM FeSO_4_·7H_2_O] supplemented with 40 mM acetamide and 12 g/l agar was used. *P. aeruginosa* cells were harvested from an LB plate, washed twice, the optical density (OD_600_) adjusted to 1.0 and inoculated onto BSM + acetamide plates.

To construct the conditional *P. aeruginosa* mutant *ΔrsmA*/IPTG-inducible *rsmN* (*ΔrsmArsmN*_ind_) a 632-bp fragment containing *rsmN* and some upstream flanking DNA was amplified from PAO1-N genomic DNA using primers RSMNPA3 and RSMNPA4 and cloned into pBluescript to give pLT6. A 572-bp fragment containing the downstream region of *rsmN* was amplified similarly using primers RSMNPA1 and RSMNPA2 to give pLT5. The plasmid pLT5 was linearized (EcoRI, BamHI) and *lacI*^Q^P_tac_ (from pME6032, EcoRI, BamHI) was introduced to give pLT7. The omega cassette (2.0 kb) was excised from pHP45Ω (BamHI) and cloned into pLT7 (cut with BamHI and dephosphorylated) to give pLT8. This plasmid was digested (EcoRI, XhoI) and the 632-bp fragment containing *rsmN* from pLT6 introduced to give pLT9. The final construct was subcloned into pDM4 (XhoI, XbaI) to give the suicide plasmid pLT10. This was mobilized into *P. aeruginosa* PAZH13 (*rsmA* deletion mutant) by mating with *E. coli* S17-1 λ*pir* to give after double homologous recombination, the conditional *rsmN* strain PALT13 (PAZH13::*lacI*^Q^P_tac_-*rsmN*).

For allelic replacement of *pprB*, 1 kb of the upstream and downstream regions of *pprB* and the *aacC1* gentamicin resistance gene (∼1 kb) were amplified and ligated by nested PCR using primers PUSpprBFw1/Rv2, PDSpprBFw5/Rv6, PaacC1Fw/PaacC1pprBRv4 and PUSpprBFw7/PDSpprBRv8. The resulting product was cloned into pGEM-T easy vector (Promega), linearized with SacI and transformed into strains PAO1-N and PALT13 by electroporation to achieve allelic replacement (20) generating the strains PAMR2 and PAMR3 respectively.

To evaluate the ability of *rsmN* to restore the biofilm-related phenotypes of *ΔrsmA, ΔrsmN* and *ΔrsmArsmN*_ind_ mutants and to control expression of translational fusion reporters, *rsmN* was expressed in *trans* using the pME6000-based plasmid pHS2, which carries a 0.47-kb insert with the wild type *rsmN* gene (18).

### Construction of plasmid expressing 6xHisRsmN, protein purification, RNA extraction and sequencing

Primers pMR4F/R, which introduce an *N*-terminal hexahistidine tag as well as EcoRI and ClaI sites, were used to amplify by PCR and clone *rsmN* into the IPTG-inducible expression vector pME6032 to generate pMR4. For the extraction of RsmN-bound RNA, recombinant 6xHisRsmN was expressed via pMR4 in wild type *P. aeruginosa* PAO1-L. 10 ml from an overnight culture of PAO1-L/pMR4 grown at 37°C were added to 190 ml of LB and incubated at 37°C with shaking until the culture reached an OD_600_ 0.6 (∼3 h). 1 mM IPTG was added and the culture incubated for a further 5 h, after which (a) 3 × 300 μl aliquots were collected for total RNA extraction and (b) the remaining culture used for 6xHisRsmN-RNA complex purification using the Ni-NTA Fast Start Kit (Qiagen) following manufacturer's instructions. The 6xHisRsmN protein–RNA was eluted with 3 × 1 ml of elution buffer and concentrated to 300 μl using 3 kDa Amicon Ultra-4 centrifugal filters (Millipore), before splitting into three aliquots.

Total RNA (a) and of 6xHisRsmN-bound RNA (b) samples were extracted using an RNeasy Mini Kit (Qiagen) and treated with Turbo DNase (Ambion) to eliminate any genomic DNA. 5 μg of RNA from each technical replicate were used for the preparation of six RNA-Seq libraries. Ribosomal RNAs were depleted from the samples using the Ribo-Zero Magnetic kit (Epicentre). Strand-specific cDNA libraries were then generated using NEBNext Ultra Directional RNA Library Prep kit for Illumina (New England BioLabs) and amplified following the TruSeq ChIP Sample Preparation Protocol. RNA sequencing was performed using MiSeq system (Illumina). To validate this method a similar preliminary experiment was carried out using two technical replicates to produce four cDNA libraries, without strand information.

### RsmN-bound RNA identification

The data processing pipeline is detailed in the Supplementary Figure S1. RNA-Seq from the libraries were mapped to the genomic sequence of PAO1-L, using a sequence derived from the PAO1-UW reference taking into account the known *rrnA/rrnB* chromosomal inversion (21) and the inclusion of RGP42 (22), using Bowtie2 tool (23) to produce .bam files. Inclusion of the large chromosomal inversion in the PAO1-UW reference sequence to match that of the PAO1-L subline used in this study was essential to map the transcripts and for the correct normalisation of strand-specific data. Each .bam file was then converted to two strand-specific .wig files, which list the number of times that each nucleotide has been mapped on the chromosome, using the bash script Bam2Wig.sh (Bedtools v2.17.0). A total of 12 .wig files were therefore generated representing a snapshot of the transcriptome of the bacterial cells grown under the conditions used and at the time of sampling, together with RNA specifically associated with RsmN at that same moment.

The .wig files obtained were first inspected with Artemis Genome Browser (24) and all were found to contain usable data. Consistency within the technical replicates was very high as revealed by principal component analysis (data not shown), although values varied in their ranges, reflecting the robustness of deep-sequencing technology but revealing the need to normalize the data. As technical replicates were homogeneous the data was merged by adding the values of the corresponding .wig files, reducing the number of files from 12 to 4. Despite the ribodepletion step carried out before sequencing, visualization of the data with Artemis revealed the presence of strong signals corresponding to ribosomal RNA operons. As this would affect normalization and subsequent analysis, signals attributable to transcripts originating from the *rrnA, rrnB, rrnC* and *rrnD* operons were given a value of zero for each of their nucleotides in the .wig files. To normalize the values of each .wig file, these were divided by their corresponding averages.

RsmN-bound RNAs were a subset of the total RNA extracted at the time of sampling; hence, dividing the normalized values of the RsmN-bound files by the total RNA files generates an ‘enrichment index’ file, in which a value >1 would correspond to a transcript bound by the protein. An exception occurred when there were no reads for a given nucleotide in the total RNA set (a transcript below detection levels in the total RNA samples) while there were reads for the RsmN-enriched sample, in which case the ratio would be undefined. Although these could potentially represent RNAs transcribed at very low levels under the experimental conditions used that could nevertheless exhibit strong affinities for RsmN, their occurrence was very rare and therefore given a value of 0.

To facilitate visualization of the data, the logarithm of the ratio values for each nucleotide multiplied by an arbitrary factor (×100) were saved into two final files corresponding to RsmN-enrichment indexes from positive and negative strands. Data was then visually analyzed with Artemis and transcripts with enrichment indexes of 2.55 or greater (RNAs 3.5-fold more abundant in the RsmN-bound fractions than in the total RNA) were selected as RsmN targets. Data from the preliminary experiment processed the same way was also used for RsmN target identification, with strand information being provided by the final experiment. The data discussed in this publication have been deposited in NCBI’s Gene Expression Omnibus (25) and are accessible through GEO Series accession number GSE94113.

### Translational reporter fusions

To construct *fhA1, tssA1* and *pelA*’-‘*lux* translational fusions, ∼0.5-kb fragments generated from PAO1-N chromosomal DNA by PCR with primers MiniCTXluxFha1Fw/Rv, MiniCTXluxTssa1Fw/Rv and MiniCTXluxPelAFw/Rv were cloned into XcmI-cut miniCTX-*lux*(Gm^r^) plasmid by *in vitro* homologous recombination using a Gibson assembly kit (New England BioLabs). Plasmids obtained were mobilized from *E. coli* S17-1 λ*pir* and mini-CTX elements inserted in the chromosome of PAO1-N Wild-type (WT) and the *ΔrsmN, ΔrsmA* and *ΔrsmArsmN*_ind_ mutants by mating. To semi-quantify c-di-GMP levels the plasmid P*cdrA*::*gfp*^S^ was mobilized into PAO1-N Wild-type (WT) and the *ΔrsmN, ΔrsmA* and *ΔrsmArsmN*_ind_ mutants respectively. Clones with active fusions were selected and analyzed for bioluminescence or fluorescence output activity over growth in LB at 37°C using a 96-well plate TECAN Genios Pro multifunction microplate reader.

### SDS-PAGE and western blotting

Protein profiles of culture supernatants of strain PAO1-N and its isogenic *ΔrsmA* and *ΔrsmN* single and double mutants were examined by treating 900 μl of cell-free overnight culture supernatants with 100 μl of trichloroacetic acid as previously described (26). Aliquots from these samples were then boiled for 10 min at 95°C and run on 12% Bis-Tris NuPAGE gels (NOVEX^®^). Trypsin digestion and LC–MS analysis of protein bands of interest for identification were performed at the Protein Nucleic Acid Chemistry Laboratory, University of Leicester, UK.

To inspect autolysis in *ΔrsmA* and *ΔrsmN* single and double mutants as well as *pprB* mutants, supernatant proteins were run on a 10% polyacrylamide SDS gel and transferred to nitrocellulose (0.2 μm, BioRad). The primary antibody (rabbit) against the cytoplasmic protein RpoS (26) was diluted 1:10,000 in 20 ml blocking solution (PBS with 0.5% Tween 20 and 5% non-fat dried skimmed milk powder) and incubated with the membrane for 1 h at room temperature. The secondary antibody anti-rabbit-HRP (Sigma) was diluted 1:2,000 in blocking solution and incubated with the membrane for 1 h at room temperature. The membrane was washed 3 × 5 min and once for 15 min in PBS with 0.5% Tween 20 before developing the blot. The membrane was dried and incubated in the Pierce^®^ ECL Western Blotting Substrate (Thermo Scientific). Blots were exposed to HyperfilmTM chemiluminescence film (GE Healthcare, Amersham Biosciences) with the use of Carestream^®^ Kodak^®^ autoradiography GBX developer/replenisher and fixer/replenisher solutions (Sigma-Aldrich).

### Electrophoretic mobility shift assays

The pET-28b(+) expression system (Novagen) was used to express 6xHis-tagged RsmN and RsmA in *E. coli* C41(DE3). Overnight cultures (10 ml) of C41 (DE3) harboring the expression plasmids were used to inoculate LB (1 L) containing the appropriate antibiotic. The culture was incubated with shaking (37°C, 200 rpm) until the OD_600_ reached 0.6–0.9 (∼3 h), at which point, the production of recombinant proteins was induced by the addition of IPTG (0.3 mM). The induced culture was incubated overnight with shaking (30°C, 200 rpm, ∼16 h). The cells were harvested by centrifugation and the cell pellet was stored at −80°C until required. 6xHis-fusion proteins were purified using Ni-NTA Fast Start Kit (Qiagen) following manufacturer's procedure.

DNA template corresponding to the target gene was amplified by PCR using primers that incorporated a T7 promoter at the 5′ end and a 17-nt extension at the 3′ end (Supplementary Table S1). The purified PCR product was used for RNA synthesis *in vitro* using the MAXIscript T7 kit (Life Technologies). The RNA obtained was visualized using a method described previously (27) consisting of the hybridization of an ATTO700-labeled DNA primer to the 3′ extension of the RNA. RsmA and RsmN were incubated with target gene RNA (5 nM) in 1 × binding buffer (10 mM Tris–Cl pH 7.5, 10 mM MgCl_2_, 100 mM KCl), 0.5 μg/μl yeast RNA (Life Technologies), 7.5% (v/v) glycerol, 0.2 units SUPERase In RNase Inhibitor (Life Technologies). Binding in the absence or presence of unlabeled competitor RNA (0.1–0.9 μM to achieve 20- to 180-fold excess) was carried out for 30 min at 37°C. Then Bromophenol Blue was added (0.01%, wt/vol) before immediate electrophoresis on 6% (w/v) non-denaturing polyacrylamide TBE gel (47 mM Tris, 45 mM boric acid, 1 mM EDTA, pH 8.3) at 4°C. Imaging was performed using a 9201 Odyssey Imaging System (LI-COR Biosciences). Image analysis and the apparent dissociation constants (Kd averages ± standard deviations) obtained for each EMSA experiment were estimated three times using Image Studio V5.0 and GraphPad Prism V7 software.

### Bacterial competition assay

To evaluate the H1 type VI secretion system prokaryotic killing capability of PAO1-N as well as *ΔrsmN* and *ΔrsmA* single and double mutants, an *in vitro* bacterial competition assay was performed using *E. coli* DH5α expressing β-galactosidase as the prey strain (28). PAO1-N strains and prey were co-incubated as spots on LB agar for 5 h at 37°C and resuspended in TSB broth. Samples were serially diluted 0 to 10^6^ × and plated in triplicate on LB plates containing X-gal (40 μg/ml). After 24 h incubation at 37°C blue color colonies were counted to quantify the survival of LacZ-positive *E. coli*.

### Alginate assay

Alginate production was determined directly from the supernatant fraction of planktonic *P. aeruginosa* strains grown in LB at 37°C 200 rpm for 14 h, using the carbazole assay (29) and alginate from *Macrocystis pyrifera* as a standard (5–120 μg/ml).

### Biofilm flow cell assay

To investigate biofilm formation by *P. aeruginosa*, a BioFlux microfluidics system (Fluxion Biosciences) was used following manufacturer's instructions and literature (30). Strains were grown overnight at 37°C, 200 rpm. The next day strains were sub-cultured and incubated in the same conditions for 6 h and the OD_600_ adjusted to 0.05. The microfluidic plates used were BioFlux 48 well plates 0–20 dyn/cm^2^. The microfluidic chambers in the plate were primed from the ‘in’ well with 100 μl 10% (v/v) LB until the fluid had filled the first circle of the ‘out’ well, 70 μl of the diluted strains were added to the ‘out’ well. The strains were then pumped from the ‘out’ chamber for 2 s at a speed of 2 dyn/cm^2^ and flow was stopped for 30 min at 37°C to allow bacterial attachment. 1 ml of pre-warmed 10% (v/v) LB supplemented with 125 nM Syto-9 (Fisher scientific) was added to the ‘in’ well, and flow started again at 0.5 dyn/cm^2^, 37°C for 14 h.

The biofilms formed were imaged using a confocal microscope (Zeiss LSM 700), at 20× magnification taking Z-stacks (∼0.92 μm) of the bottom layer of the chambers. Syto-9 excitation and emission wavelengths were set to 483 and 500 nm respectively. Biomass quantification from image stacks of biofilms was done with Comstat2 software (31) (www.comstat.dk) using default parameters.

### Congo red binding assay

The colony morphologies and pigmentation of the *ΔrsmA* and *ΔrsmN* single and double mutants were noted after growth on Congo red + Coomassie blue agar plates. Samples from overnight bacterial cultures were collected, OD_600_ was adjusted to 1.0 and spotted (5 μl) onto 1% tryptone agar plates supplemented with Congo red (40 μg/ml) and Coomassie brilliant blue (20 μg/ml). Plates were incubated at room temperature for up to 6 days.

### Extracellular DNA quantification

To quantify autolysis in strain PAO1-N and its isogenic *ΔrsmA, ΔrsmN* and double *ΔrsmArsmN*_ind_ mutants as well as *pprB* mutants, extracellular DNA (eDNA) release was assessed in 18 h LB cultures (37°C, 200 rpm). After measuring the OD_600_ of the cultures, 1 ml of each was centrifuged and the cell-free supernatant filtered (0.22 μm). To precipitate eDNA, 450 μl of clear supernatant were then mixed in an Eppendorf with 50 μl of 3 M sodium acetate pH 5.2 and 1 ml of ice-cold 100% ethanol, left at −20°C for 30 min and centrifuged 30 min at 4°C, 13,000 rpm. The pellet was then washed once with 70% (v/v) ethanol, air-dried and resuspended in 45 μl of molecular biology grade water. The concentration of eDNA was then measured with a NanoDrop™ spectrophotometer, and values normalized to the corresponding OD_600_. Each measurement was carried out in triplicate for each strain, and means compared with those obtained for the wild type.

## RESULTS

### Genome-wide mapping of the RsmN regulon

To identify genes potentially regulated by RsmN through direct interaction with their corresponding transcripts, His-tagged RsmN was expressed in *P. aeruginosa* PAO1-L and purified bound to its RNA targets. These were extracted and subjected to deep-sequencing to characterize the RsmN regulon under the growth conditions used. As a control, total RNA was extracted from the same strain for data normalization.

A total of 503 transcripts were enriched above the set threshold in the RNA samples obtained by co-purification with RsmN compared with total RNA (Supplementary Figure S2 and Supplementary Table S2). Of these, 393 could be linked to annotated mRNAs or sRNAs classified by their predicted functions, which are diverse (Supplementary Table S2), while 110 transcripts could not be associated with the nearest annotated genes (e.g. divergently transcribed or transcribed as antisense RNAs). These could potentially correspond with as yet unannotated open reading frames or non-coding RNAs. Of the mRNAs identified, 199 encode proteins that have already been characterised. These include transcripts from virulence genes involved in acute infection, for instance those coding for a T3SS used by *P. aeruginosa* to inject toxins directly into host cells such as the effector protein ExoT, the effector translocator protein PopD (32) and HtpG, a heat shock protein required for secretion of ExoS (33). Another important mRNA bound to RsmN was that for the acute virulence regulator gene *vreI*, encoding an Extra Cytoplasmic Function (ECF) sigma factor mediating the induction of the Hxc T2SS gene cluster (34).

Transcripts from genes contributing to chronic biofilm infections were also bound to RsmN including *cgrA, cgrC* and *cupB6*, encoding regulators and components of the CupA and B secretion systems involved in the formation of fimbrial appendages that facilitate biofilm formation (35,36). Also genes related to EPS production such as *pelA* and *pelD* involved in Pel exopolysaccharide biosynthesis (37) and *mucA*, a negative regulator of alginate biosynthesis (38). Other attachment and biofilm-related RNAs included members of the TCSs *creC, mifR* and *pprB* (39–41). Furthermore, RsmN bound several transcripts coding for a phosphodiesterase (PDE) (*bifA*) and the diguanylate cyclases (DGCs) *siaD* and *roeA*, which inversely regulate levels of the second messenger c-di-GMP that controls the switch from motile to sessile lifestyles in *P. aeruginosa* (42–44). A number of transcripts were identified coding for the island 1 type VI secretion system (H1-T6SS) which kills bacterial competitors inhabiting the same ecological niche and promotes antibiotic tolerance in biofilms (45). These include structural components of the injectisome (TagT1, TssA1, TssB1, TssC1 and TagJ1) as well as its assembly and activation apparatus (TagS1, PpkA and FhA1) (46,47). Moreover, transcripts of genes involved in H2-T6SS (IcmF2) and H3-T6SS (HsiC3) that translocate trans-kingdom effectors (48) were also found bound to RsmN.

RsmN also bound to the small regulatory RNA CrcZ, involved in the carbon catabolite repression mechanism that enables *P. aeruginosa* to utilise preferential carbon sources for maximal growth (49). In addition, RsmW, a recently described Rsm-like sRNA that is upregulated in nutrient-limiting conditions, in biofilms, and at higher temperatures was identified as a putative RsmN target (50). As anticipated, the GacS/GacA-controlled RsmY sRNA previously shown to bind *in vitro* to this protein (18,19) was also identified. Even though RsmZ was also expected and this transcript was clearly present in the total RNA samples, it was not identified here as an RsmN-bound RNA, perhaps reflecting the lower affinity of RsmN for RsmZ than for RsmY *in vivo*.

### RsmN post-transcriptionally represses *fhA1* gene expression and exerts a negative impact on T6SS

**Figure 1.**
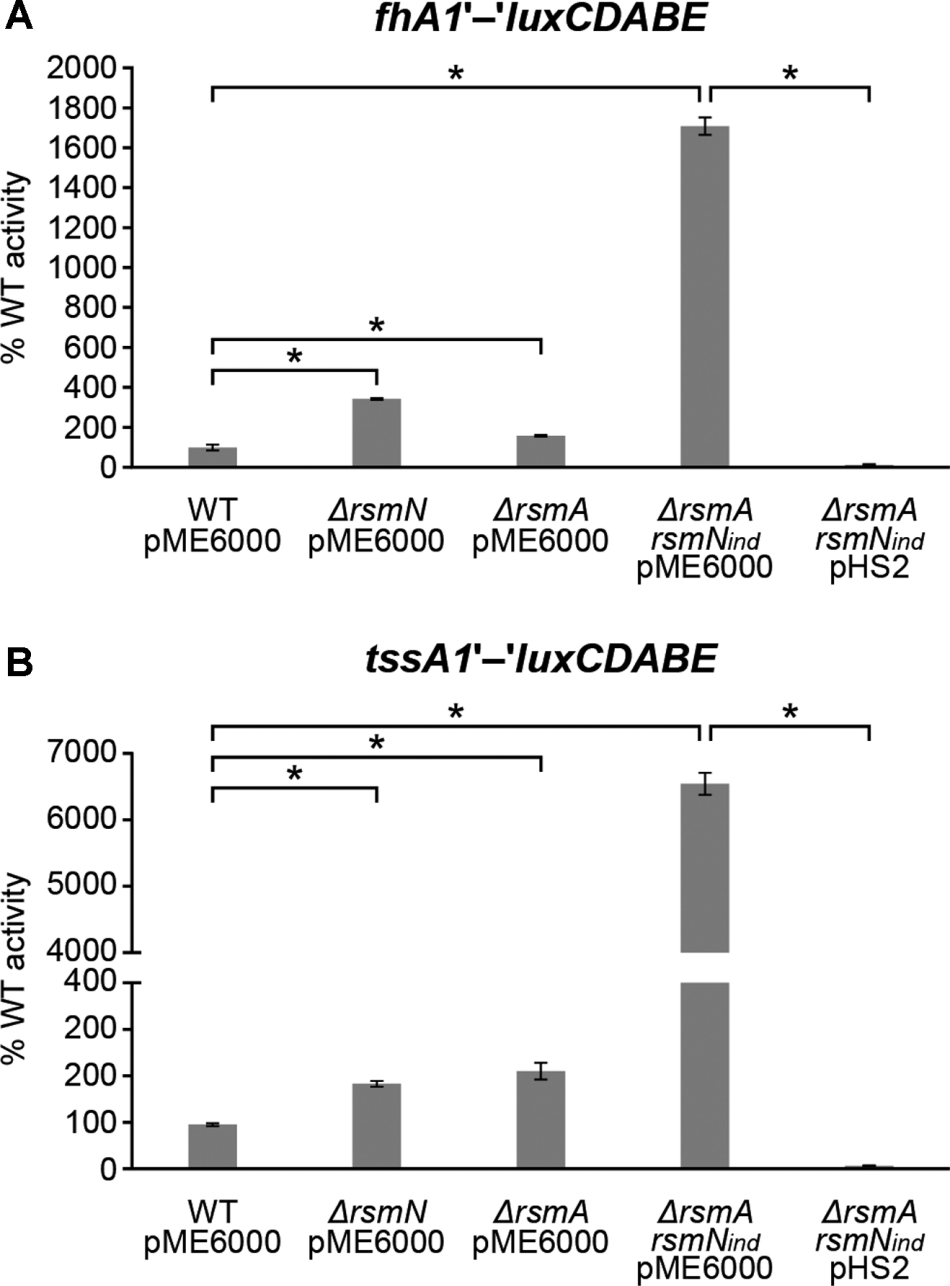
Effect of *rsmN* and/or *rsmA* mutations on H1-T6SS gene translation. Wild type PAO1-N and the *rsm* mutants carrying the translational fusions (**A**) *fhA1*’-‘*luxCDABE* and (**B**) *tssA1*’-‘*luxCDABE* in their chromosomes were transformed with a vector control (pME6000) or pHS2 (expressing *rsmN*). Values given are averages from three different cultures ± standard deviation and correspond to the area under the curve (AUC) derived from plotting relative light units normalized to culture density (RLU/OD_600_) over time (24 h), and as percentage of the corresponding activity obtained in the WT (set at 100%). Statistical differences between group means were determined by one-way ANOVA tests (*p < 0.05).

To determine whether RsmN directly regulates H1-T6SS partly through the control of *fhA1* expression, we first constructed a translational fusion to the *lux* operon (*fhA1*’-‘*luxCDABE*) using the miniCTX system and introduced it onto the *P. aeruginosa* PAO1-N wild-type (WT) and the *ΔrsmN, ΔrsmA* and *ΔrsmArsmN*_ind_ mutant chromosomes respectively. As a control, a similar construct was made for *tssA1* (*tssA1*’-‘*luxCDABE*), as RsmN is known to bind this T6SS structural component (19). In agreement with previously published results for *tssA1*, while a modest de-repression of *fhA1* expression was recorded in *ΔrsmN* and *ΔrsmA* mutants, de-repression in a double *ΔrsmArsmN*_ind_ mutant was significantly enhanced compared with the WT and single mutants. Moreover, expression of *rsmN in trans* in the double mutant restored the repression of the *fhA*1’-‘*lux* translational fusion (Figure 1A). Similar results were obtained for the *tssA1* fusion (Figure 1B). These data confirm that RsmN is a negative regulator of T6SS in *P. aeruginosa* PAO1 and that this regulation takes places through the control of different secretion machinery components.

**Figure 2.**
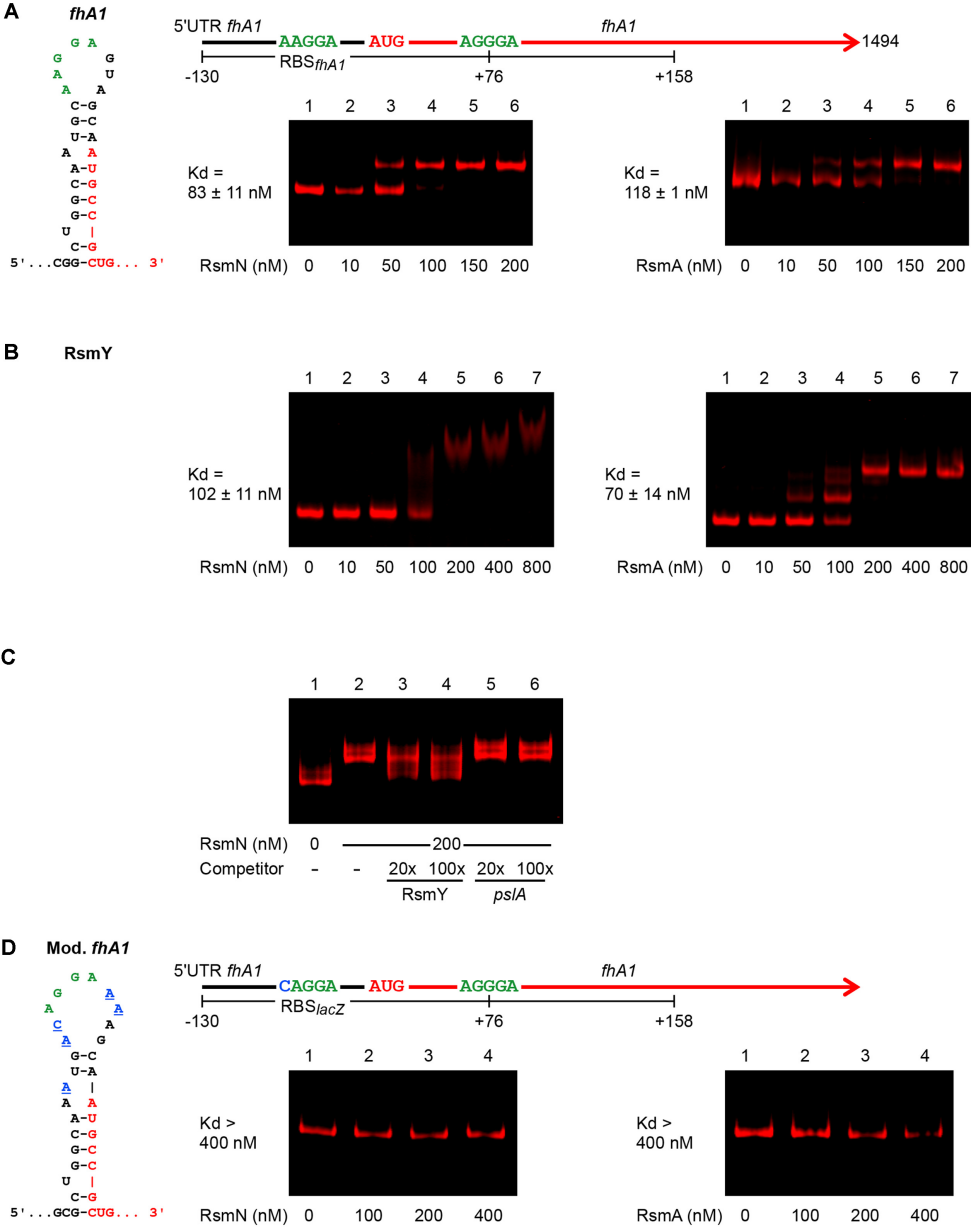
Binding of RsmN and RsmA to *fhA1* mRNA. (**A**) Diagram of the leader sequence (black) and open reading frame (red) of *fhA1* showing the putative Rsm-binding sites (green), the predicted hairpin structure formed at the SD of *fhA1*, and the 288-nt RNA transcribed *in vitro* for the binding assay (nucleotides –130 to +158 relative to the *fhA1* start codon). EMSA results indicate comparable binding by RsmN and RsmA to this RNA, with an apparent slightly higher affinity for the former. (**B**) Binding of RsmN and RsmA to full-length RsmY transcribed *in vitro*. In this case RsmA appears to have a higher affinity and multiple binding compared to RsmN. (**C**) Binding of RsmN to *fhA1* mRNA in the presence of 20- and 100-fold excess RsmY (specific) and *pslA* (non-specific) competitor RNAs. Under these conditions the electrophoretic mobility of the *fhA1* mRNA is partially left in the unbound state in the presence of RsmY while it remains unaltered with the non-specific competitor. (**D**) Map of leader sequence (black) and *fhA1* open reading frame (red) with a modified, non-RsmA-regulated SD derived from that of *lacZ* (blue) and binding assays with RsmN and RsmA. Underlined characters in the predicted hairpin structure indicate nucleotides that differ from the wild type *fhA1* sequence (Mod*fhA1*). EMSAs were carried out using fluorescently labeled RNA (5 nM). Incubated in the absence (lane 1) or presence of increasing concentrations of purified RsmN or RsmA protein (A, lanes 2–6; B, lanes 2–7; D, lanes 2–4), or in the presence of 200 nM RsmN and either 20- or 100-fold excess of competitor RNA, as indicated. Dissociation constants (*K*_d_ averages ± standard deviation) derived from these results are indicated.

To confirm the binding specificity of RsmN for the *fhA1* transcript, an electrophoretic mobility shift assay (EMSA) was carried out using a 6xHis-tagged RsmN and a 3′-labeled fluorescent RNA corresponding to the leader of *fhA1* (Figure 2A). As controls, binding of RsmA to the *fhA1* RNA (Figure 2A) and of RsmN and RsmA to the full-length RsmY RNA (Figure 2B) were also tested. Both proteins were able to retard the mobility of *fhA1* RNA in native gel electrophoresis, demonstrating direct binding of both regulators to the *fhaA1* mRNA (Figure 2A and Supplementary Figure S5). This binding could be reduced by adding RsmY but not by adding a non-specific competitor RNA derived from *pslA* (Figure 2C and Supplementary Figure S5). Consistent with previous studies, RsmY complexes showed different migration patterns when bound to RsmA or RsmN: RsmA, at 50 nM or greater, produced up to three shifted bands, probably corresponding to multiple molecules of RsmA bound to RsmY (19,51,52). RsmN-RsmY complexes however, did not show such a clear laddered pattern, although a similar shift was observed for the higher molecular weight band for both proteins (Figure 2B), similar to what has recently been reported (51). In contrast to RsmY, a single shift for both RsmA and RsmN was observed for *fhA1* RNA. This could be explained by the existence of a single Rsm binding site or a dimer binding to two sites on the RNA used (Figure 2A).

Analysis of the nucleotide region around the Shine-Dalgarno sequence (SD) of the *fhA1* mRNA revealed the presence of an RsmA binding motif (AAGGA) in a stem–loop structure overlapping with the ribosome bind site (RBS) (Figure 2A). To determine whether this loop is required for the RsmN and RsmA-mediated repression of *fhA1* translation, a third translational reporter was constructed by substituting the *fhA1* native SD with the non RsmA-regulated SD of *lacZ*. As result, no repression by RsmN and RsmA was observed providing further evidence that both proteins negatively regulate *fhA1* expression at the post-transcriptional level and likely through the recognition of the AAGGA motif (details of the substitutions appear in Figure 2D and results in Supplementary Figure S3). To investigate this further, an RNA with the above SD substitution was generated to test the binding of RsmN/A using EMSA. Both proteins failed to bind to the modified *fhA1* transcript with the *lacZ* SD (Figure 2D), supporting the possibility that RsmA and RsmN may prevent *fhA1* mRNA translation initiation through binding to this region.

A comparison of protein profiles from supernatants of WT, *ΔrsmA, ΔrsmN* and *ΔrsmArsmN*_ind_ cultures showed differences in their secreted protein profiles. In particular, a band with a molecular weight of approximately 17 kDa was consistently detected in the supernatant of the double *ΔrsmArsmN*_ind_ mutant and its abundance was reduced upon RsmN expression (Supplementary Figure S4). The identity of the protein corresponded to the T6SS effector Hcp1 (PA0085, with 96% probability) confirming the negative impact of RsmN on T6SS expression (19). To determine whether the translational de-repression of T6SS components observed in *ΔrsmN* and *ΔrsmA* mutants corresponded with an increased killing capability of *P. aeruginosa* PAO1-N, a bacterial competition assay was performed using *E. coli* DH5α as the prey strain. Quantification of *E. coli* survival indicated that *ΔrsmN, ΔrsmA* and *ΔrsmArsmN*_ind_ strains kill more prey than the WT PAO1-N (Supplementary Table S3). Furthermore, prey survival levels of the *ΔrsmArsmN*_ind_ strain complemented with *rsmN* are similar to WT levels, confirming the negative impact on RsmN on T6SS expression.

### RsmN and RsmA specifically bind to *mucA* RNA and inhibit alginate biosynthesis

To assess the impact of RsmN on genes involved in modulating biofilm development, the binding of RsmN to the *mucA* transcript (PA0763; Supplementary Table S2) was determined. The *mucA* gene codes for an inner membrane-bound antagonist of the sigma factor AlgU that induces transcription of the TCS AlgZR. Together, AlgU and AlgZR induce the transcription of genes encoding the biosynthetic enzymes required for alginate production (38). This exopolysaccharide is an extracellular matrix component of *P. aeruginosa* biofilms, which in certain environments makes an important contribution to biofilm community architecture (53–55). In the lungs of individuals with cystic fibrosis (CF), alginate-overproducing variants arise frequently due to mutations in *mucA* (56), conferring a mucoid phenotype that contributes to antibiotic tolerance and reduces host immune clearance of *P. aeruginosa* from the respiratory tract (57).

**Figure 3.**
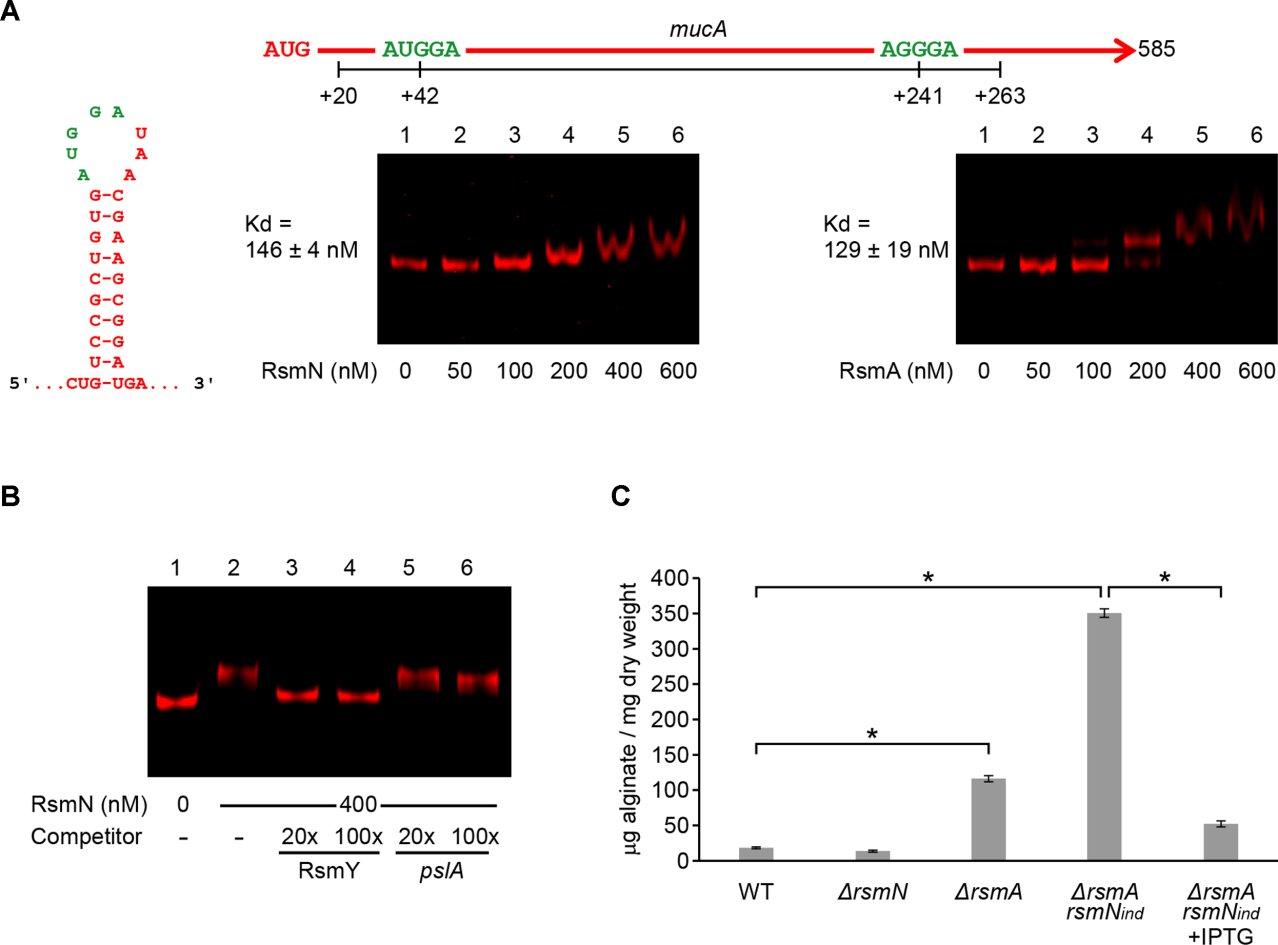
Binding of RsmN and RsmA to *mucA* mRNA and their control of alginate production. (**A**) Map of *mucA* open reading frame showing the putative Rsm-binding sites (green), predicted hairpin structure and binding assays of RsmN and RsmA to a 240-nt RNA transcribed *in vitro* corresponding to an internal region of the *mucA* open reading frame (nucleotides +23 to +263 relative to the start codon). Fluorescently labelled RNA (5 nM) was incubated in the absence (lane 1) or presence of increasing concentrations of RsmN or RsmA (lanes 2–6). Dissociation constants (*K*_d_ averages ± standard deviation) from these results are indicated. (**B**) Binding of RsmN at 400 nM to *mucA* mRNA in the presence of RsmY (specific) and *pslA* (non-specific) competitor RNAs carried out as in Figure 2C. In this case, while the electrophoretic mobility of the *mucA* mRNA remains unaltered in the presence of non-specific competitor, it is completely left in the unbound state in the presence of RsmY. (**C**) Alginate production measured using the carbazole assay from the supernatant fraction of PAO1-N wild type (WT) and the *ΔrsmN, ΔrsmA* and *ΔrsmArsmN*_ind_ mutants (without and with IPTG induction of *rsmN*) grown in LB at 37°C for 14 h. Values given are averages from three different cultures ± standard deviation. Statistical differences between group means were determined by one-way ANOVA tests (*p < 0.05).

To determine whether RsmN effectively binds to the *mucA* transcript, an EMSA was carried out using RNA corresponding to an inner region of the *mucA* open reading frame transcribed *in vitro* and including two putative RsmA-binding motifs. One of which (AUGGA) is present in a predicted single-stranded region of a stem-loop structure. An EMSA with RsmA was also carried out to determine whether *mucA* is an RsmN-specific target. Despite *mucA* not having previously been described as an RsmA target, EMSAs indicate that both RsmN and RsmA bind *mucA* mRNA with similar affinities (observed *K*_d_: 129–150; Figure 3A and Supplementary Figure S5), and therefore, the gene coding this anti-AlgU sigma factor is a shared target for both post-transcriptional regulators. Specificity of the binding was challenged with RsmY and *pslA* RNA as specific and non-specific competitors; while RsmY prevented the band shift, the addition of *pslA* RNA had no effect (Figure 3B and Supplementary Figure S5). To identify the binding site for RsmN, two *mucA* RNAs, each carrying one of the motifs were synthesized and binding assessed. No binding was detected for either RNA using EMSA (data not shown), a result in agreement with recent work indicating that RsmN may require two GGA sites for binding (8).

Since RsmN and RsmA binding motifs are located within the *mucA* coding region and as MucA is a cytoplasmic membrane protein, the construction of a translational reporter fusion to this gene was not feasible. Therefore, to investigate the impact of RsmN and RsmA on MucA, alginate production was quantified in the WT, *ΔrsmN, ΔrsmA* and *ΔrsmArsmN*_ind_ PAO1-N strains. Although the effect of the *rsmN* mutation on alginate production was indistinguishable from the WT, the *ΔrsmArsmN*_ind_ double mutant produced significantly higher yields of alginate than either the WT (19-fold) or *ΔrsmA* single mutant (3-fold). Conversely, IPTG-induced expression of *rsmN* in the double *ΔrsmArsmN*_ind_ mutant greatly reduced alginate production (Figure 3C). These results indicate that both RsmN and RsmA negatively affect alginate production, probably through a positive effect on *mucA* mRNA stability and translation yields.

### RsmN negatively regulates the production of Pel exopolysaccharide

Alginate is generally synthesised in low levels by strains isolated from environments other than the CF lung. In non-mucoid strains, Psl and/or Pel are the predominant EPSs of the matrix of mature biofilms. These biofilm matrix constituents support surface colonization by facilitating aggregation and adherence. The relative importance of both EPSs seems to be strain-dependent. While Psl is the primary EPS scaffold component of *P. aeruginosa* PAO1 biofilms, other strains incapable of Psl production such as *P. aeruginosa* PA14 rely on Pel (54). Psl and Pel exhibit different spatial organization within the biofilm matrix and their location corresponds with the different phases of biofilm development. Psl gives biofilms structural firmness and architecture localizing in the periphery of mature biofilm microcolonies (58) and is also essential for the early stages of cell adherence to biotic or abiotic surfaces (54). Pel is thought to cross-link with eDNA to support and stabilize the interior and stalk of biofilm microcolonies. Moreover, in Psl deficient strains, Pel can compensate for the absence of Psl in the periphery of cell aggregates (37). The production of different types of EPSs expands the antibiotic tolerance as well as increasing the persistence of *P. aeruginosa* biofilms under changing environmental conditions.

**Figure 4.**
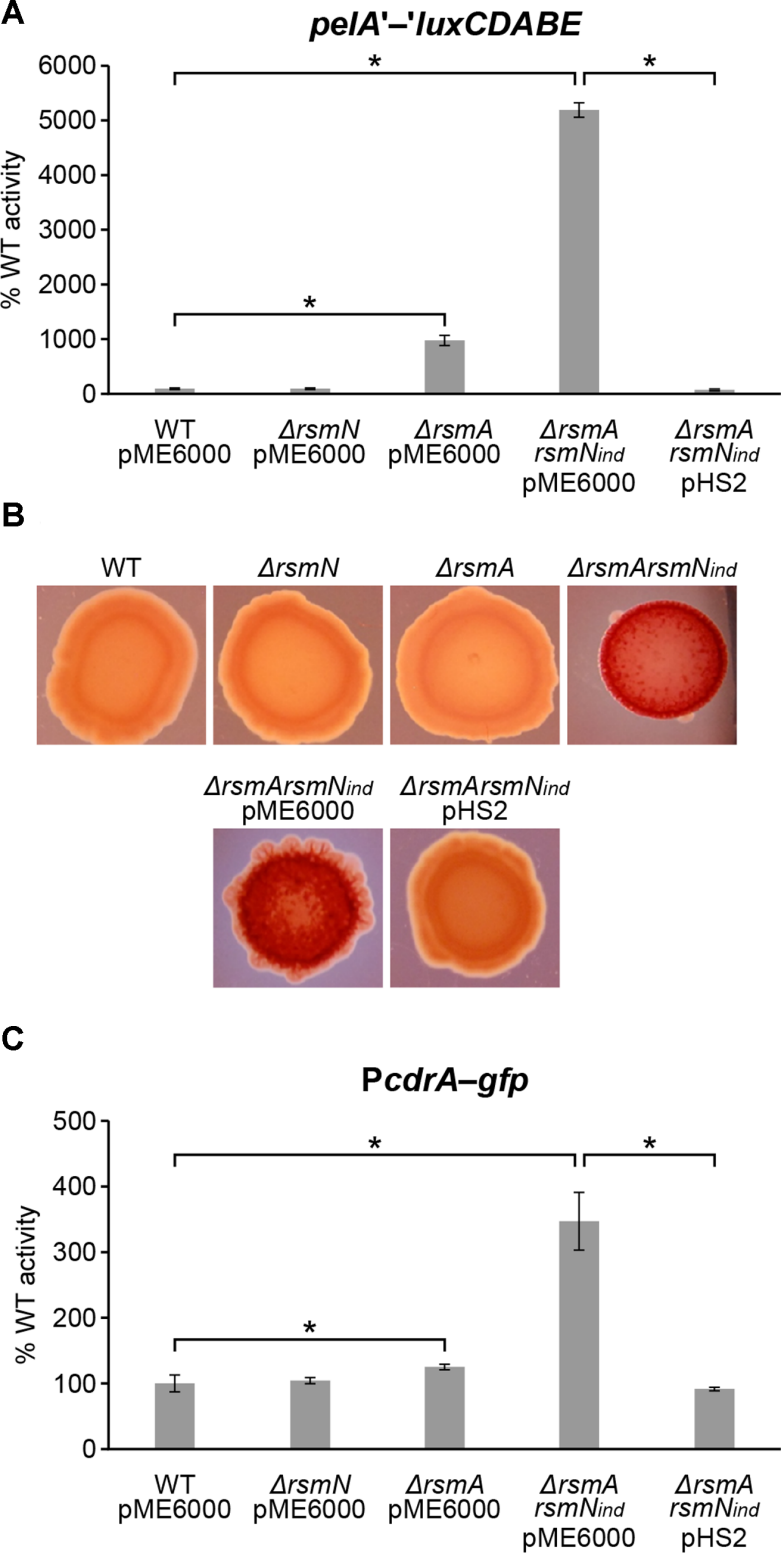
Effects of *rsmN* or/and *rsmA* mutations on *pelA* translation, colony morphology and c-di-GMP levels. (**A**) WT PAO1-N and the indicated mutants carrying a *pelA*’-‘*luxCDABE* chromosomal translational fusion were transformed with the vector control (pME6000) or pHS2 (expressing *rsmN*). Values were obtained as described in Figure 1. (**B**) Colony morphology and appearance of *P. aeruginosa* PAO1-N strains grown on 1% tryptone 1% agar plates supplemented with Congo red (40 μg/ml) and Coomassie brilliant blue (20 μg/ml) at room temperature for 6 days. Deep red colonies of double mutant *ΔrsmArsmN*_ind_ + pME6000 (vector control) were complemented with *rsmN* via pHS2 and restored to wild type. (**C**) Effect of *rsmN* or/and *rsmA* mutations on c-di-GMP levels. WT PAO1-N and the indicated mutants carrying the transcriptional fusion P*cdrA-gfp*^S^ which reports intracellular c-di-GMP levels were transformed with a vector control (pME6000) or pHS2 (expressing *rsmN*). Reported values are averages from three different cultures ± standard deviation and correspond to the area under the curve (AUC) derived from plotting relative fluorescence units normalized to culture density (RFU/OD_600_) over time (24 h), and as percentage of the corresponding activity obtained in the WT (set at 100%). Statistical differences between group means were determined by one-way ANOVA tests (*p< 0.05).

In contrast to RsmA, RsmN is unable to bind to the *pslA* transcript required for Psl biosynthesis (19). Our data confirms this given that we did not find any *psl* transcripts bound by RsmN. However, we noted that transcripts for the Pel biosynthesis genes (*pelA* and *pelD*) were bound to RsmN, suggesting that it may specifically regulate Pel rather than Psl. To investigate this, we introduced a *pelA*’-‘*luxCDABE* translational fusion onto the chromosome of the PAO1-N WT and the *ΔrsmN, ΔrsmA* and *ΔrsmArsmN*_ind_ mutants respectively. A significantly enhanced translational de-repression in the double *ΔrsmArsmN*_ind_ mutant with respect to the isogenic single mutants was observed (Figure 4A). Conversely, expression of *rsmN in trans* repressed the *pelA*’-‘*lux* translational fusion in the double mutant (Figure 4A), indicating that RsmN is a negative regulator of Pel.

To further validate this finding, we visualized EPS production in the WT, *ΔrsmA, ΔrsmN* and *ΔrsmArsmN*_ind_ PAO1-N strains on agar plates supplemented with Congo red (CR). The WT, *ΔrsmA* and *ΔrsmN* mutant strains grew as smooth light orange-red colonies on CR agar (Figure 4B), while the *ΔrsmArsmN*_ind_ double mutant showed an enhanced CR staining phenotype with rough, deep red colonies. Additionally, complementation of the *ΔrsmArsmN*_ind_ mutant with plasmid pHS2 reverted the phenotype to wild type (Figure 4B) confirming the negative impact of RsmN on Pel production.

### RsmA and RsmN negatively control c-di-GMP levels

Given that biofilm formation is regulated by c-di-GMP signalling through EPS production (43,44,59) and that DGC and PDE genes (*bifA, siaD* and *roeA*) were identified among RsmN targets (Supplementary Table S2), we assessed this second messenger in the WT, *ΔrsmA, ΔrsmN* and *ΔrsmArsmN*_ind_ strains using a *gfp*^S^-based transcriptional fusion to the *cdrA* promoter (60). As shown in Figure 4C, the *ΔrsmArsmN*_ind_ mutant showed high levels of *cdrA* expression, well above of that of the WT and single mutant strains, consistent with elevated c-di-GMP levels. Complementation of *rsmN in trans* reduced these levels to those of the WT strain, further supporting the hypothesis that RsmN impacts on EPS production and hence biofilm formation directly via post-transcriptional control of *pel* and indirectly by reducing c-di-GMP levels.

### RsmN post-transcriptionally regulates *pprB* expression and cell lysis

**Figure 5.**
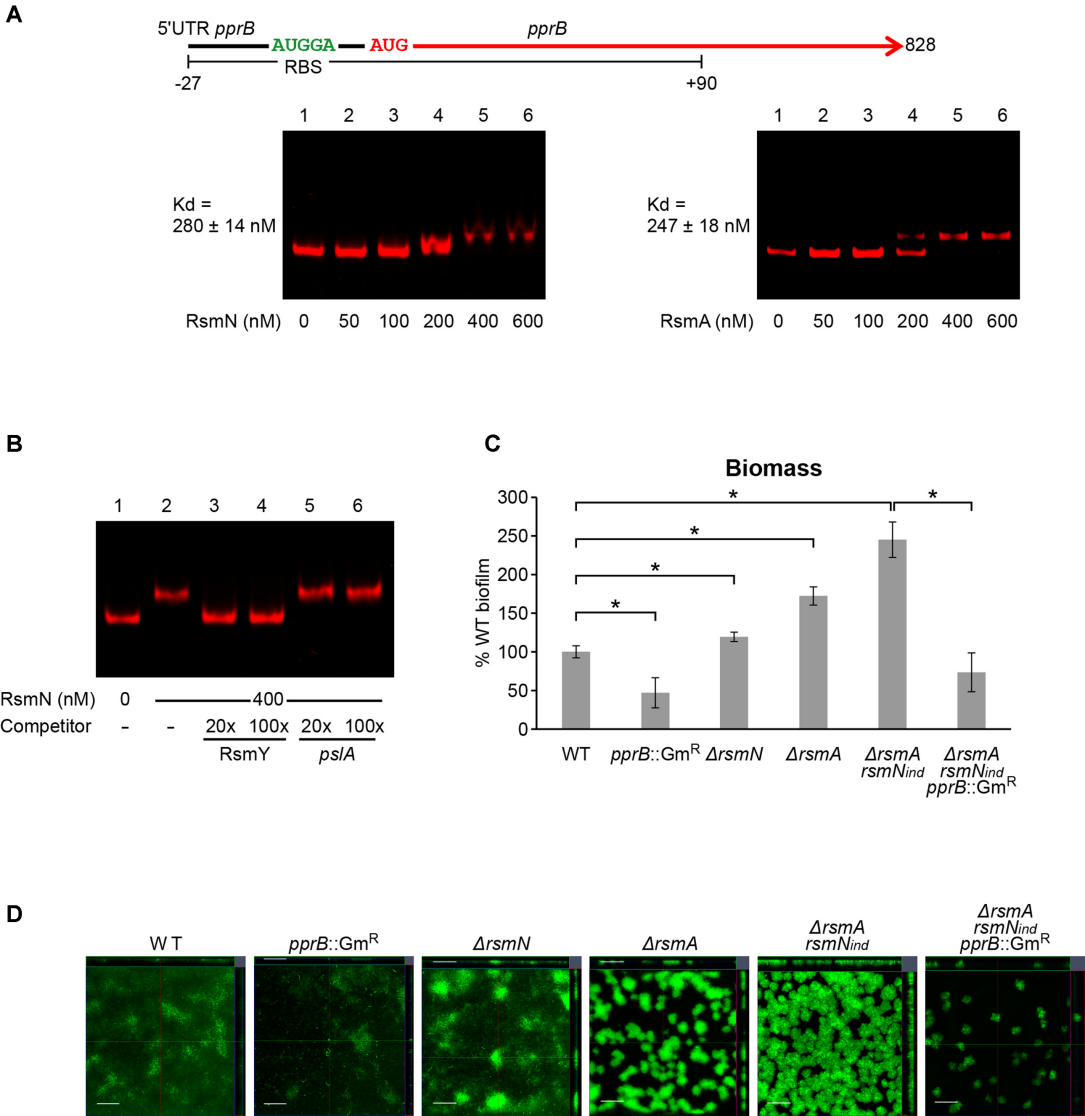
Binding of RsmN and RsmA to *pprB* mRNA and control of biofilm development by the Rsm system. (**A**) Map of the leader (black) and open reading frame (red) of *pprB* showing the putative Rsm binding site (green). Binding assays of RsmN and RsmA to a 117-nt RNA transcribed *in vitro* for the binding assay (nucleotides –27 to +90 relative to the *pprB* start codon). Fluorescently labelled RNA (5 nM) was incubated in the absence (lane 1) or presence of increasing concentrations of RsmN or RsmA (lanes 2–6). Dissociation constants (Kd averages ± standard deviation) from these results are indicated. (**B**) Binding of RsmN at 400 nM to *pprB* mRNA in the presence of RsmY (specific) and *pslA* (non-specific) competitor RNAs carried out as in Figure 2C. The electrophoretic mobility of the *pprB* mRNA remains unaltered in the presence of non-specific competitor, while it is completely left in the unbound state in the presence of RsmY. (**C**) Biofilm biomass was quantified using Comstat2 from four image stacks of biofilms formed by the different strains. Values reported are normalized to percent WT activity (set at 100%). Statistical differences between group means were determined by one-way ANOVA tests (*p< 0.05). (**D**) Confocal microscopy images of biofilms grown for 14 h in microfluidic chambers using a BioFlux system. Scale bars correspond to 50 μm. Bacteria were incubated at 37°C and stained with Syto-9 fluorescent dye for visualization and quantification.

The mRNA coding for PprB, which plays a role in biofilm formation, was discovered bound to RsmN. PprB is a TCS member that relies on the BapA adhesin, CupE chaperone usher type fimbriae, Flp Type IVb pili and eDNA to trigger a hyper-biofilm phenotype in an EPS-independent manner (39). PprB has also been associated with antibiotic hyper-susceptibility, decreased virulence in acutely infected *Drosophila*, and reduced cytotoxicity toward various cell types that is linked to reduced T3 secretion (39). To determine whether either or both RsmN and RsmA bind to the *pprB* transcript, EMSAs were carried out using RNA corresponding to a leader that includes a predicted RsmA binding motif (AUGGA) located in a single-stranded region of a stem-loop overlapping with the RBS. The results obtained show that *pprB* is a shared RsmN and RsmA target, as both proteins bound specifically to the same region of the *pprB* RNA (Figure 5A and Supplementary Figure S5). As with *mucA* mRNA, the band shift obtained with *pprB* could be completely abolished by the presence of RsmY but not by the non-specific competitor *pslA* RNA (Figure 5B and Supplementary Figure S5).

Using an indirect crystal violet staining method, a *P. aeruginosa* PA14 *ΔrsmN* mutant was reported to form similar biofilms to those of the parent whereas *ΔrsmA* and *ΔrsmAN* mutants both showed increased biofilm formation with more robust biofilms in the latter (19). To investigate whether the effect was due to the influence of these mutations on *pprB* expression, the impact of a triple *ΔrsmArsmN*_ind_/*pprB* mutant on biofilm formation under controlled flow conditions was compared with that of *pprB, ΔrsmN, ΔrsmA* and *ΔrsmArsmN*_ind_ mutants. The *pprB* mutant showed significantly reduced biofilm formation compared with the WT strain, confirming the involvement of this response regulator in attachment and biofilm development (39,61) (Figure 5C and D). In contrast, the *ΔrsmA* and *ΔrsmArsmN*_ind_ mutants developed biofilms with higher numbers of microcolonies after 12 h of growth that were more discrete in the *ΔrsmArsmN*_ind_ double mutant (Figure 5D).

Interestingly, the *ΔrsmN* single mutant showed an intermediate phenotype between the flat and undifferentiated biofilm of the WT and the microcolony organization of the *ΔrsmA* mutant biofilm. This suggested that RsmN has a negative impact on microcolony formation under the conditions tested. This phenotype was reversed in the *ΔrsmN* mutant complemented with plasmid pHS2 (Supplementary Figure S6). While microcolonies in the *ΔrsmA* mutant were clearly visible after 8 h of biofilm development, those formed by the *ΔrsmN* mutant only became apparent after 12 h (data not shown). Remarkably, a significant reduction in biofilm formation (biomass) was observed when *pprB* was deleted in the *ΔrsmArsmN*_ind_ mutant (Figure 5C), suggesting that *pprB* is required for the negative impact of RsmA and RsmN on microcolony formation. Furthermore, expression of *rsmN* from pHS2 in the *ΔrsmArsmN*_ind_ mutant significantly reduced biofilm biomass, surface area and thickness to levels similar to those found in the WT, although its properties were not fully restored and appeared to confer an intermediate phenotype (Supplementary Figure S7).

PprB overexpression results in significant induction of the pseudomonas quinolone signalling (PQS) system (39) and consequently eDNA release via cell lysis (62). We therefore assayed the eDNA content of supernatants from PAO1-N *ΔrsmA* and *ΔrsmN* single and double mutants as well as the *pprB* mutant. Indeed, eDNA levels were substantially greater in the *ΔrsmArsmN*_ind_ double mutant whereas *rsmN* expression *in trans* reduced eDNA towards WT levels. The release of eDNA was however inhibited when *pprB* was mutated in the *ΔrsmArsmN*_ind_ strain (Supplementary Figure S8). Moreover, immunoblotting allowed detection of high levels of the cytoplasmic sigma factor RpoS (26) in supernatants from the *ΔrsmArsmN*_ind_mutant. Induction of *rsmN* expression in this mutant reduced the levels of extracellular RpoS to those of the WT (Supplementary Figure S9), suggesting that PAO1 cells undergo autolysis in the absence of both RsmA and RsmN possibly through post-transcriptional regulation of *pprB* expression.

### RsmN positively regulates CrcZ an sRNA involved in carbon catabolite repression

**Figure 6.**
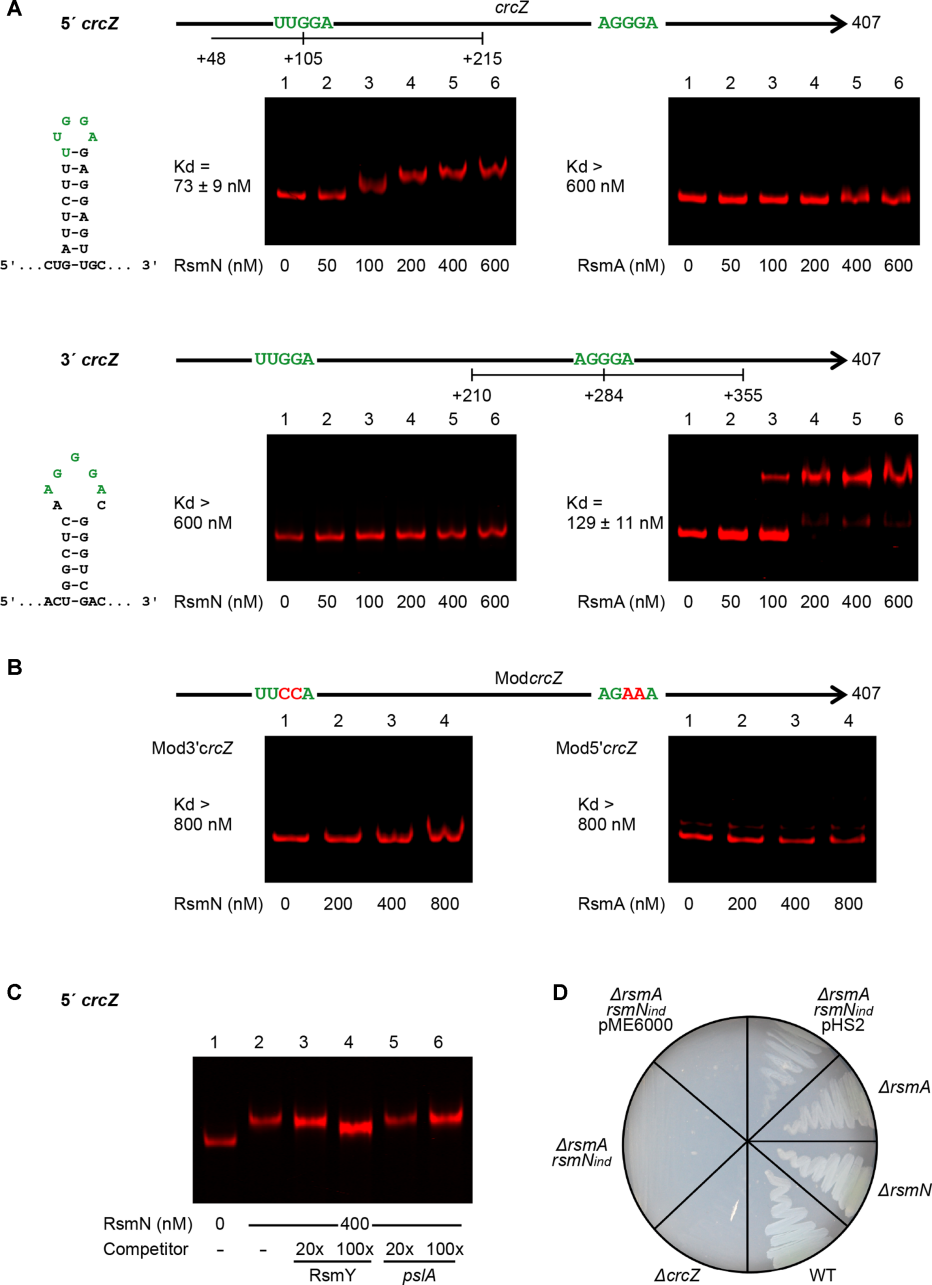
Binding of RsmN and RsmA to specific moieties of CrcZ regulatory RNA and control of carbon assimilation by the Rsm system. (**A**) Map of CrcZ RNA showing the putative Rsm binding sites (green), predicted hairpin structures formed around these sites and EMSAs of RsmN and RsmA on RNAs transcribed *in vitro* corresponding to the 5′ (nucleotides +48 to +215 of the transcript) or 3′ (nucleotide +210 to +355) moieties of CrcZ. Dissociation constants (Kd averages ± standard deviation) from these results are indicated. (**B**) Map of modified CrcZ (Mod*crcZ*) with nucleotide substitutions (red) in the Rsm binding sites (green) and EMSAs of RsmN and RsmA on RNA moieties of Mod*crcZ* transcribed *in vitro* as in (A). Fluorescently labelled RNAs (5 nM) were incubated in the absence (lane 1) or presence of increasing concentrations of RsmN or RsmA (A lanes 2–6; B lanes 2–4). (**C**) Binding of RsmN at 400 nM to the 5′ moiety of CrcZ in the presence of RsmY (specific) and *pslA* (non-specific) competitor RNAs carried out as in Figure 2C. The electrophoretic mobility of 5′ CrcZ is partially left in the unbound state in the presence of RsmY while it remains unaltered with the non-specific competitor. (**D**) Growth of *P. aeruginosa* PAO1-N strains on BSM minimal medium agar supplemented with 40 mM acetamide as the sole carbon source, after 24 h of incubation at 37°C.

The RNAseq results showed that CrcZ bound to RsmN. The role of CrcZ in carbon catabolite repression (CCR) (49) adds a further dimension to the contribution of RsmN to *P. aeruginosa* metabolism. To investigate the regulatory role of RsmN, full-length CrcZ was transcribed *in vitro* and tested for both RsmN and RsmA binding using an EMSA. The mobility shift obtained with full-length CrcZ RNA showed two bands when using native gel electrophoresis making it difficult to visualise (data not shown). Analysis of the sequence of CrcZ revealed a single ANGGA motif in the 3′ region of the RNA (AGGGA), while no typical RsmA-binding motifs were present in the 5′ region of the transcript. Instead, a UUGGA motif predicted to be in an un-paired region of a stem-loop structure was identified (Figure 6A). Consequently, CrcZ was separated into two regions, corresponding to the 5′ and 3′ moieties of the CrcZ transcript with each carrying one of the putative binding motifs. RsmN/A binding to each region was assessed independently.

Surprisingly, RsmN and RsmA both bind to CrcZ but to different regions. While RsmN only bound to the first 5′ region of CrcZ, RsmA solely shifted the band corresponding to the 3′ region of this sRNA (Figure 6A and Supplementary Figure S5), a shift that could be partially reversed with the specific competitor RsmY but not with the non-specific *pslA* RNA (Figure 6C). To determine whether RsmA and RsmN bind to CrcZ through interactions with the above motifs, variants of the same RNA with substitutions in both motifs and without affecting the predicted stem-loop structures (AGGGA to AGAAA and UUGGA to UUCCA) were constructed and transcribed *in vitro*. No band shifts were detected for either RsmA or RsmN in EMSAs using the mutated RNAs demonstrating that binding requires the presence of the motifs (Figure 6B).

These results are indicative of cross-talk between the CCR and Rsm regulatory systems. To further investigate this, we assessed the growth of PAO1-N WT, *ΔcrcZ, ΔrsmN, ΔrsmA* and *ΔrsmArsmN*_ind_ mutants in BSM minimal medium supplemented with the poor carbon source acetamide. When a non-preferred substrate is the sole carbon source, expression of *crcZ* increases resulting in the sequestration of Hfq protein, the release of ribosome binding sites and the translation of catabolic genes (49). Therefore, if RsmA/N interactions alter CrcZ function we would expect to observe changes following growth on acetamide. In common with the WT strain, the *ΔrsmA* and *ΔrsmN* mutants both grew on BSM plates with acetamide as the sole carbon source. However, the double *ΔrsmArsmN*_ind_ mutation hindered growth on acetamide, resembling the *crcZ* mutant phenotype. Growth could be restored by expressing *rsmN in trans*, suggesting a positive effect of RsmN on CrcZ function and a contribution to catabolite repression (Figure 6D).

## DISCUSSION

Since the discovery of CsrA in *E. coli* (4), members of the CsrA/RsmA family have been identified in diverse bacterial species, including the Pseudomonads (63), which possess 2–5 homologues (e.g. *P. syringae* (64)). CsrA/RsmA proteins have attracted considerable attention as they facilitate rapid bacterial adaptation to diverse environmental conditions and regulate virulence (16,17). Despite *P. aeruginosa* initially being an apparent exception with only a single *rsmA* allele, a second unique CsrA/RsmA homologue was recently discovered (18).

Previous studies characterised the *P. aeruginosa* RsmA regulon indirectly using transcriptome analysis (3,65). Here, sequencing of RNAs co-purifying with RsmN was chosen to identify directly genome-wide targets. Furthermore, the ability of RNASeq to generate nucleotide level information on strand-specific transcript abundance and to identify transcripts corresponding to unannotated open reading frames and uncharacterized non-coding RNAs was also considered advantageous. Although 110 such potential RsmN-binding transcripts of known or predicted function were detected, we have not yet characterised these further and only focused on three of known function involved in biofilm formation and chronic infections (*fhA1, mucA* and *pprB*) and primary metabolism/catabolite repression (CrcZ; Supplementary Figure S10). Binding of RNAs from these selected candidates was confirmed via EMSAs, and although the enrichment values from RNAseq depend on the regions considered, a correlation between these and the observed Kd was noted (Supplementary Table S4; Supplementary Figure S10).

**Figure 7.**
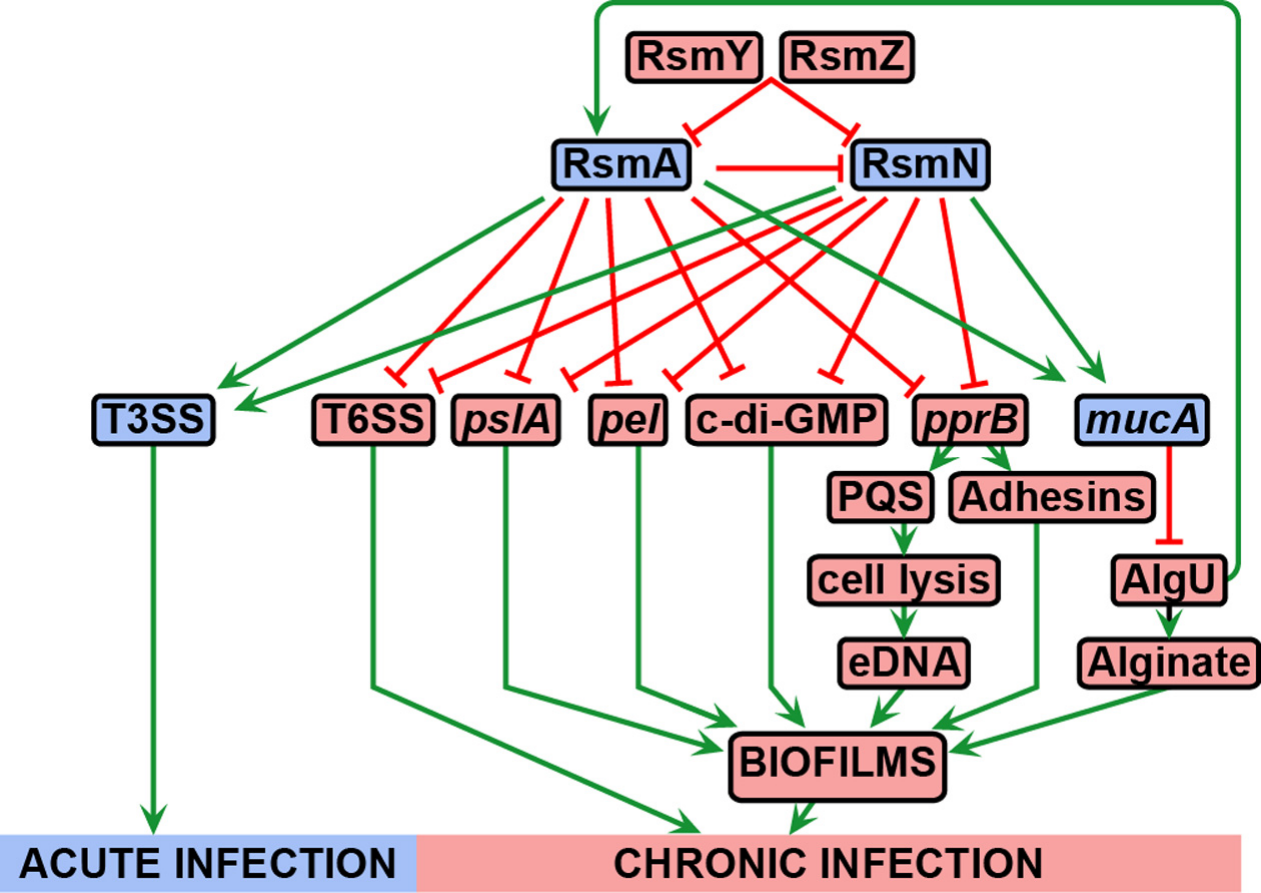
Regulatory network involving RsmA and RsmN governing *P. aeruginosa* infections. Acute infection is characterised by the direct and indirect repression of several elements required for the formation of biofilms and the establishment of chronic infections, a mechanism in which the Rsm system participates at the post-transcriptional level. Type III and VI secretion systems (T3SS and T6SS, respectively) are inversely regulated by the system between the two infection lifestyles. Elements which increased abundances/activities are characteristic of acute or chronic infections are shaded in blue or pink, respectively, and elements acting positively or negatively on others are represented by arrowheads and bars, respectively.

The robustness of our approach is supported by our finding of RsmN-bound transcripts previously described for RsmA (Supplementary Table S2) (3,8,12,65–67). This partial overlap was expected and likely to expand as further RsmA targets are verified. Validation of these targets supports a central role for RsmN in the ‘switch on’ of traits required for acute infections and the ‘switch off’ of those for chronic infections (Figure 7). For example, RsmA positively controls T3SS structural components and effectors involved in enhancing cytotoxicity during acute infections (3,67), including the ExoT transcript that we found bound to RsmN. In contrast, and in common with RsmA, RsmN represses *fhA1* (Figure 1A), the product of which is a key scaffolding protein of the H1-T6SS that confers a fitness advantage on *P. aeruginosa* via the killing of other prokaryotes (9). Accordingly, the increased levels of the T6SS effector Hcp1 (haemolysin co-regulated protein PA0085) found in supernatants from *ΔrsmArsmN*_ind_ mutant cultures were reduced to wild type by expression of *rsmN* (Supplementary Figure S4). Furthermore, transcripts of genes involved in H2-T6SS (IcmF2) and H3-T6SS (HsiC3) secretion systems, that translocate trans-kingdom effectors (48), were also found bound to RsmN (Supplementary Table S2). As RsmA regulates the three T6SSs in *P. aeruginosa* (9) these results strongly suggest that RsmA and RsmN regulation of these secretion systems overlaps.

Our results show that RsmN impacts broadly on biofilm maturation, a phenotype closely associated with chronic infections. This control seems to be mainly in partnership with RsmA and is exerted at multiple levels resulting in a net negative effect (Figure 7). In particular, we observed that RsmN negatively impacts on EPS production (Figures 3C, 4A and B). RsmN directly binds to the coding region of the *mucA* transcript (Figure 3A and B; Supplementary Figure S5). MucA sequesters the sigma factor AlgU, which via AlgZR, activates the expression of alginate biosynthetic genes (38) impacting on both biofilm architecture and antimicrobial resistance (53–55). Although prevention of ribosome binding is the most intensively investigated mode of action of CsrA/RsmA proteins, a synergistic effect on de-repression of alginate production in the *ΔrsmArsmN*_ind_ double mutant compared with the WT and single mutants was observed in this study (Figure 3C). This suggests that both RsmN and RsmA positively impact on *mucA* expression possibly via mRNA stabilisation. In fact CsrA enhances *fhlDC* and *moaA* expression in *E. coli* by either protecting their mRNAs from 5′ end-dependent RNase E cleavage or by binding to a potential MoCo-dependent riboswitch (68,69). Moreover, a recent study (70) reported transcript stabilisation by CsrA acting as an anti-terminator of the PPP/glycolysis *gap* operon mRNA in *Legionella pneumophila*. Thus, the CsrA/RsmA family bind to mRNAs and impact on gene expression by at least four different mechanisms, three of which have been exemplified with enhanced expression of the target genes.

A link between MucA/AlgU/AlgZR and the Rsm regulatory system has been noted before, as AlgZR inhibits *exsA* expression, and consequently T3SS production, partially by inducing *rsmY* and *rsmZ* and reducing the abundance of free RsmA (71). AlgU directly upregulates *rsmA* transcription (72) such that enhanced expression of *mucA* by RsmA and RsmN should reduce AlgU activity which in turn negatively impacts on *rsmA* expression so increasing RsmN production. This suggests a mechanism for self-modulation of *rsmA* and *rsmN* expression and therefore virulence determinants (Figure 7).

The *pelA* transcript required for Pel biosynthesis was found bound to RsmN suggesting that this regulator directly controls the *pel* operon (Figure 4A and B). In contrast, RsmA directly controls *pslA* expression suggesting differential regulation of the two EPSs by the two RNA binding proteins (10,19). Given that elevated *pel* expression can compensate for Psl deficiency, and that these two EPSs display some grade of functional overlap (37,54), the direct regulation of *pel* mRNA stability or translation by RsmN may facilitate better control of this redundant EPS biosynthesis and sustain the prevalence of Psl in the biofilm matrix (54).

Transcripts from the DGCs and PDE RoeA, SiaD and BifA with known roles in early biofilm formation were present among the RsmN targets (Supplementary Table S2), strengthening the established links between the Rsm and c-di-GMP regulatory networks (42,66,73–75). RoeA promotes Pel production to enhance surface adhesion via type IV pili (76) whereas BifA modulates Pel production and swarming motility inversely. The loss of BifA function leads to hyperbiofilm formation and an inability to swarm (43), a phenotype positively regulated by RsmN (18). SiaD is required for *P. aeruginosa* cell aggregation after exposure to sodium dodecyl sulfate (SDS) by regulating the expression of a core set of genes including those coding for CupA fimbriae (42). Moreover, SiaD was essential for purified Psl polysaccharide to stimulate c-di-GMP production (77). The P*cdrA*::*gfp*^S^ reporter fusion data suggest that the elevated c-di-GMP in the *ΔrsmArsmN*_ind_ double mutant coud be reduced back to parental by expressing *rsmN in trans*, indicating that it also negatively affects c-di-GMP levels and therefore signalling (Figure 4C). Interestingly, RsmN also binds to the transcript coding for the c-di-GMP receptor PelD, which mediates c-di-GMP-dependent regulation of Pel biosynthesis (59). This suggests that RsmN may also modulate c-di-GMP signalling by controlling the production of this c-di-GMP receptor.

Other RsmN bound biofilm regulatory gene transcripts included *pprB*, a finding we validated via EMSAs (Figure 5A and B; Supplementary Figure S5). Deletion of *pprB* resulted in a significant reduction in biofilm formation in the robust biofilm-forming *ΔrsmArsmN*_ind_ double mutant suggesting that *rsmA* and *rsmN* mutations may increase *P. aeruginosa* surface colonization partly through post-transcriptional regulation of *pprB* (Figure 5C and D). PprB is a response regulator that directly and positively controls BapA adhesion expression, type IVb Flp pili and chaperone usher CupE fimbriae which contribute to biofilm formation (39). The PQS system is also upregulated by PprB and contributes to cell lysis and eDNA release (62). Consistent with this, the cytoplasmic sigma factor RpoS was detectable in the *ΔrsmArsmN*_ind_ mutant culture supernatant (Supplementary Figure S9) suggesting that *P. aeruginosa* cells undergo autolysis in the absence of both RsmA and RsmN. Furthermore, substantially more eDNA release was found in *ΔrsmArsmN*_ind_ mutant supernatants compared with the WT (Supplementary Figure S8). These suggest that RsmA and RsmN control biofilm development through cell lysis and e-DNA release via the PprB and PQS systems.

Although *rsmA* deletion has greater impact than *rsmN* deletion, regulation by the latter becomes more apparent when both genes are disrupted. A possible explanation for this would be that, under the experimental conditions used, regulation of shared targets is mainly controlled by RsmA. This is supported by the finding that RsmA represses RsmN translation (19) and that RsmN generally appears to have lower affinity than RsmA for overlapping targets. An exception could be *fhA1*, which was observed to have slightly greater affinity for RsmN (Figure 2A and Supplementary Figure S5) and where expression of *fhA1*’-’*lux* fusion in the *ΔrsmA* mutant was slightly less de-repressed than in the *ΔrsmN* mutant (Figure 1A). Likewise, a recent study reported that RsmN had reduced affinity towards target RNAs with respect to RsmA despite sharing a preference for an artificial binding sequence obtained using the SELEX approach (8). Therefore, for many transcripts an *rsmN* deletion would exert only a modest effect on their stability and translation given the presence of RsmA. Conversely, in a *ΔrsmA* mutant background, RsmN would only partially replace RsmA due to its reduced affinity for the same transcripts. In a *ΔrsmArsmN*_ind_ double mutant a synergistic effect on translation repression/de-repression occurs. A synergistic effect when disrupting multiple *rsmA* alleles in pseudomonads has previously been observed, as deletions of either *rsmA* or *rsmE* only partially upregulated expression of genes coding for antifungal secondary metabolites and exoenzymes in *Pseudomonas protegens* CHA0, whereas a double *ΔrsmAE* deletion strongly increased expression of these common target genes (64).

The hypothesis that RsmA is the primary regulator of shared targets with RsmN due its higher affinity for the transcripts is in agreement with the synergistic effects of a double *ΔrsmArsmN*_ind_ mutation on the expression of shared targets when restricted to transcripts where RsmA and RsmN both bind to the same GGA-containing motifs. However, this is not always the case, as found with RsmA and RsmN binding to different regions of a sRNA such as CrcZ (Figure 6A and B). In this case, *ΔrsmA* and *ΔrsmN* single mutants showed identical phenotypes with respect to growth on acetamide as the sole carbon source, and only when both regulators were mutated was a significant phenotypic change observed (Figure 6D). This is a striking result since, despite binding to the same transcript, RsmN appears to interact at a different location and at a site with a sequence divergent from the established conserved RsmA RNA binding motif (Figure 6A and B). This suggests that there may be an additional specificity for alternative binding motifs determined by the structural differences between RsmN and RsmA (18,19,51). To verify this at a global level, Rsm-RNA binding studies at a higher resolution, as recently described for *Salmonella enterica* (78), would be needed as *in vitro* selection of consensus sequences for RsmA/N binding are poor predictors of RsmN targets.

The binding of RsmA/N to CrcZ suggests cross-talk between catabolite repression and the Gac/Rsm systems. The effect of Rsm on *P. aeruginosa* metabolism is modulated by a signalling pathway that controls transcription of RsmY and RsmZ sRNAs via the TCS GacS/GacA. The environmental signal(s) that triggers this pathway has not been identified in *P. aeruginosa*, however, it appears likely that carboxylated products of catabolism may be sufficient to trigger signal transduction via GacS/GacA (79,80). Moreover, transcription of *crcZ* is induced by CbrAB during growth on less-preferred carbon sources such as acetamide and reduced in the presence of carboxylated nutrients such as succinate (81). Thus, both Gac/Rsm and CCR systems appear to be modulated by carboxylates. Furthermore, new findings establishing direct and indirect connections between catabolite repression and the Csr (Rsm) system in *E. coli* (82) support the link found in this study. CrcZ may interact with RsmA and RsmN and repress their actions similarly to RsmY/Z to further co-ordinate the output of CCR and the Gac/Rsm regulatory systems. RsmA and RsmN together could stabilize CrcZ allowing *P. aeruginosa* to grow on less-preferred carbon sources. However, further experimental research will be required to dissect mechanistically the link between Rsm and CCR.

Although the additional RsmN-bound transcripts remain to be validated, no full-length transcripts exclusively bound by RsmN were identified. However, given that RsmN interacts with a different GGA motif within the sRNA CrcZ, RsmN has the potential to control a specific subset of targets even if these are associated with RsmA-controlled functions. If this is the case, it is intriguing that *P. aeruginosa* maintains two functionally redundant Rsm paralogues that probably arose via gene duplication (18). From an evolutionary perspective, only negligible selective pressure would act on the redundant new genes; consequently, changes in function and divergence due to high rates of mutation would be expected. However, although rapid divergence and loss of redundant function in gene duplicates is more frequent, redundant genes frequently remain active and unchanged (83–85).

The presence of redundant Rsm proteins that can replace or by-pass each other's activities could help bacteria achieve greater plasticity via post-transcriptional regulation and better noise control in gene expression. Alternatively, as can be extrapolated from this study, the different affinities observed for RsmA and RsmN for their targets may result in different efficiencies in the control of gene expression that could also explain the conservation of these redundant Rsm paralogues. As previously suggested for redundant regulators (86), cell functions controlled by Rsm protein duplication could profit from the sum of each protein dose. Consequently, although RsmA concentrations in the cell may vary due to noise in gene expression or apparent induction, the combined concentrations of RsmA and RsmN may be maintained in a homeostatic equilibrium.

## Supplementary Material

Supplementary Data
