# Supplementary material for: Genome-wide mapping of the RNA targets of the Pseudomonas aeruginosa riboregulatory protein RsmN
Source: Nucleic Acids Res. 2018 Apr 30;46(13):6823–40. doi: 10.1093/nar/gky324 (PMC6061880; doi:10.1093/nar/gky324)
Supplement: Supplementary Data [file gky324_supplemental_files.zip › Supplementary_Data_2018_04_10.pdf]

Genome-wide mapping of the RNA targets of the *Pseudomonas aeruginosa* riboregulatory protein RsmN

Manuel Romero, Hazel Silistre, Laura Lovelock, Victoria J. Wright, Kok-Gan Chan, Kar-Wai Hong, Paul Williams, Miguel Camara & Stephan Heeb

SUPPLEMENTARY DATA

Table of contents

page
Table S1 2-4
Figure S1 5
Figure S2 6
Table S2 7-17
Figure S3 18
Figure S4 18
Table S3 19
Figure S5 19
Figure S6 20
Figure S7 21
Figure S8 22
Figure S9 22
Table S4 23
Figure S10 24

Table S1. Bacterial strains, plasmids and oligonucleotides used in this study.

Strain, plasmid or oligonucleotide	Relevant characteristics	Reference or origin
E. coli		
C41(DE3)	Host strain for T7 RNAP-dependent gene expression	Lucigen
DH5α	Cloning strain	(87)
S17-1 λ*pir*	Strain for maintenance and mobilization of R6K replicons	(88)
P. aeruginosa		
PAO1-L	Wild type PAO1 strain, Lausanne subline	B. Holloway via D. Haas
PAO1-N	Wild type PAO1 strain, Nottingham subline	This laboratory
PAZH13	Δ*rsmA* in-frame deletion mutant, derivative of PAO1-N	(89)
PALT16	Δ*rsmN* in-frame deletion mutant, derivative of PAO1-N	(18)
PALT13	PAZH13::ΩSm/Spc-$lacI^{Q}P_{tac}$-*rsmN* (=$\Delta rsmA rsmN_{ind}$)	This study
PAMR2	*pprB*::*aacC1*, derivative of PAO1-N (=*pprB*::Gm^{R})	This study
PAMR3	*pprB*::*aacC1*, derivative of PALT13 (=$\Delta rsmA rsmN_{ind}$*pprB*::Gm^{R})	This study
PAO6679	Deletion of *crcZ* promoter, derivative of PAO1-L	(81)
Plasmids		
pBluescript II KS	Cloning vector; Ap^{r}	Stratagene
P*cdrA*::gfp^{S}	pUCP22Not-P*cdrA*-RBS-CDS-RNaseIII-*gfp*(Mut3)-T0-T1, Ap^{r} Gm^{r}; c-di-GMP reporter	(60)
pDM4	R6K-derived suicide vector; *sacBR*; Cm^{r}	(90)
pET-28b(+)::(His6-Thb-*rsmN*)	pET-28b(+)-based construct to produce RsmN 6xHis-tagged at the N-terminus in *E. coli*	(18)
pET-28b(+)::(His6-Thb-*rsmA*)	pET-28b(+)-based construct to produce RsmA 6xHis-tagged at the N-terminus in *E. coli*	(18)
pGEM-T easy	Vector for cloning PCR amplicons; Ap^{r}	Promega
pHP45Ω	Source of BamHI Ω (Sm^{R}/Sp^{R}) cassette	(91)
pHS2	pME6000 with a 0.47-kb chromosomal DNA insert carrying *rsmN*	(18)
pLT10	pDM4::*rsmNabcd*; suicide vector to insert Ω–$lacI^{Q}P_{tac}$ immediately upstream of *rsmN*, rendering it IPTG-inducible	This study
pLT5	pBluescript II KS::*rsmNa* (amplified with RSMNPA3 and RSMNPA4)	This study
pLT6	pBluescript II KS::*rsmNd* (amplified with RSMNPA1 and RSMNPA2)	This study
pLT7	pBluescript II KS::*rsmNab* (pLT5 with $lacI^{Q}P_{tac}$ cloned from pME6032)	This study
pLT8	pBluescript II KS::*rsmNabc* (pLT7 with inserted Ω–Sp cassette)	This study
pLT9	pBluescript II KS::*rsmNabcd* (pLT8 with *rsmN* fragment from pLT6)	This study
pME6000	pBBR1MCS derivative cloning vector; Tc^{r}	(92)
pME6001	pBBR1MCS derivative cloning vector; Gm^{r}	(93)
pME6032	$lacI^{Q}P_{tac}$ expression vector; pVS1-p15A shuttle vector; Tc^{r}	(94)
pMiniCTX-lux(Gm^{r})	Gentamicin-resistant variant of pMiniCTX-*lux*	Fletcher *et al.*, in preparation
pMR4	pME6032-based construct to produce RsmN 6xHis-tagged at the N-terminus in *P. aeruginosa*	This study
Oligonucleotides	Sequence (5'-3')	Usage
PUSpprBFw1	GCACGGGGATGATCGCCTTG	Allelic replacement of *pprB* with *pprB*::*aacC1*
PUSpprBRv2	TTCATCCGTTTCCACGGTGTGCGTCGCGAGGCCGGTTTGTCCAT	Ditto
PaacC1Fw	GACGCACACCGTGGAAACGGATGAA	Amplification of *aacC1* from pME6001 for the allelic replacement of *pprB* with *pprB::aacC1*
PaacC1PprBRv4	TCAGTGCACCACCGCTCCGCGCGGCGTTGTGACAATTTACCGAAC	As above
PDSpprBFw5	GCGGAGCGGTGGTGCACTGA	Allelic replacement of *pprB* with *pprB::aacC1*
PDSpprBRv6	ACATTCTCGGCGCGGATGGA	As above
PUSpprBFw7	ACCTCGATCCGCGACGTGGC	As above
PDSpprBRv8	GGGGACCATCGAACAGTTGT	As above
RSMNPA1	TATGAATTCATGGGTTTCCTGATACTCTC	Construction of suicide plasmid pLT10, EcoRI site underlined

RSMNPA2	TATCTCGAGGGCGACTCCACCAAGACC	As above, XhoI underlined
RSMNPA3	TATTCTAGACCAGGTTGAGCTGATTGAGG	As above, XbaI underlined
RSMNPA4	TATGGATCCCCTTTGGTGAATGAAATGGTGT	As above, BamH underlined
pMR4F	TATGAATTCATGCACCATCACCATCACCATGGTTTCCTGATACTCTCCCG	Construction of pMR4, EcoRI site underlined followed by 6 histidine codons, anneals *rsmN* at the 2nd codon
pMR4R	TATATCGATTCAGCCTTTCGGTGCCGTTT	Construction of pMR4, ClaI site underlined adjacent to *rsmN* stop codon.
MiniCTXluxFhA1Fw	TGTTGGTACGACCAGTTCGCAAGATAGTTAAACAGCAACTTAAGTTGAAAGTTCGAGGAACGCGGCGTCGTAC	Amplify *fha1* promoter region and clone it into miniCTXlux reporter system
MiniCTXluxFhA1Rv	TCGGGAAAGATTTCAACCTGGCCGTTAATAATGAATGAAATTTTTTTAGTGCAGTGGCGGCTGGAGACCAGGC	As above
MiniCTXluxTssA1Fw	TGTTGGTACGACCAGTTCGCAAGATAGTTAAACAGCAACTTAAGTTGAAACGGTTGAGGATGGTGCAGTGGCG	Amplify *tssA1* promoter region and clone it into miniCTXlux reporter system
MiniCTXluxTssA1Rv	TCGGGAAAGATTTCAACCTGGCCGTTAATAATGAATGAAATTTTTTTAGTCGCGGCGTCGTACTCCAGATCGTCGCC	As above
MiniCTXluxPelAFw	TGTTGGTACGACCAGTTCGCAAGATAGTTAAACAGCAACTTAAGTTGAAATTCCTCGCACGCAACTGAAG	Amplify *pelA* promoter region and clone it into miniCTXlux reporter system
MiniCTXluxPelARv	TCGGGAAAGATTTCAACCTGGCCGTTAATAATGAATGAAATTTTTTTAGTACGCAGCACGGCGATTCCTT	As above
PT73'crcZFw	TTTTCTGCAGTAATACGACTCACTATAGGCATCAGCGACCAAAGCAAT	Primer for EMSA, to amplify 3' moiety of CrcZ and add T7 promoter
P3'crcZFTRv	AAAAAAAACCCCCCCCCAAGTAGGTGCGTG	As above
P3'crcZCRv	CCCCCCCAAGTAGGTGCGTG	As above
PT75'crcZFw	TTTTCTGCAGTAATACGACTCACTATAGGTAACGACTCCAGCACAACA	Primer for EMSA, to amplify 5' moiety of CrcZ and add T7 promoter
P5'crcZFTRv	AAAAAAAACCCCCCCCCTGATGCTGCCATGATCGTC	As above
P5'crcZCRv	CTGATGCTGCCATGATCGTC	As above
PT7fha1Fw	TTTTCTGCAGTAATACGACTCACTATAGGACCCGATGTGTCTTCCCGAG	Primer for EMSA, to amplify *fha1* and add T7 promoter
Pfha1FTRv	AAAAAAAACCCCCCCCCTTAGTGCAGTGGCGGCTGGA	As above
Pfha1CRv	TTAGTGCAGTGGCGGCTGGA	As above
PT7mucAFw	TTTTCTGCAGTAATACGACTCACTATAGGAAACTCTGTCCGCTGTG	Primer for EMSA, to amplify *mucA* and add T7 promoter
PmucAFTRv	AAAAAAAACCCCCCCCCGACCGACCATCCGCCACG	As above
PmucACRv	CGACCGACCATCCGCCACG	As above
PT7pprBFw	TTTTCTGCAGTAATACGACTCACTATAGGACAAAATAAGCAAATGGAGC	Primer for EMSA, to amplify *pprB* and add T7 promoter
PpprBFTRv	AAAAAAAACCCCCCCCCGTTTTCCAGCAGTTCGCGGA	As above
PpprBCRv	GTTTTCCAGCAGTTCGCGGA	As above
PT7rsmYFw	TTTTCTGCAGTAATACGACTCACTATAGGGTCAGGACATTGCGCAGGAA	Primer for EMSA, to amplify *rsmY* and add T7 promoter
PrsmYFTRv	AAAAAAAACCCCCCCCCGCCTTTTGGGCGGGGTTTTG	As above
PrsmYCRv	GCCTTTTGGGCGGGGTTTTG	As above
pslA-T7-F	TAATACGACTCACTATAGGGACAAAGCCACTATCGACGAAT	Primer for EMSA, to amplify *pprB* and add T7 promoter
pslA-T7-R	CATGTTGTTTGCTCTGCCGA	As above
EMSAFT	*AAAAAAAACCCCCCCCC	5' ATTO700-labeled oligo

EMSA primer nomenclature: oligonucleotides that incorporate a T7 promoter at the 5' end are indicated by T7, these that incorporate a 17-nt extension corresponding to the ATTO700-labeled DNA oligonucleotide to the 3' end of template RNA are indicated by FT. Fw and Rv: forward and reverse PCR primers respectively.

References for Supplementary Table S1

87. Grant, S.G., Jessee, J., Bloom, F.R. and Hanahan, D. (1990) Differential plasmid rescue from transgenic mouse DNAs into *Escherichia coli* methylation-restriction mutants. *Proc. Natl. Acad. Sci. USA*, **87**, 4645-4649.

88. Simon, R., Priefer, U. and Pühler, A. (1983) A broad host range mobilization system for *in vivo* genetic engineering: transposon mutagenesis in Gram-negative bacteria. *Nat. Biotechnol.*, **1**, 784-791.

89. Pessi, G., Williams, F., Hindle, Z., Heurlier, K., Holden, M.T., Cámara, M., Haas, D. and Williams, P. (2001) The global posttranscriptional regulator RsmA modulates production of virulence determinants and N-acylhomoserine lactones in *Pseudomonas aeruginosa*. *J. Bacteriol.*, **183**, 6676-6683.

90. Milton, D.L., O'Toole, R., Horstedt, P. and Wolf-Watz, H. (1996) Flagellin A is essential for the virulence of *Vibrio anguillarum*. *J. Bacteriol.*, **178**, 1310-1319.

91. Prentki, P. and Krisch, H.M. (1984) In vitro insertional mutagenesis with a selectable DNA fragment. *Gene*, **29**, 303-313.

92. Maurhofer, M., Reimmann, C., Schmidli-Sacherer, P., Heeb, S., Haas, D. and Défago, G. (1998) Salicylic Acid Biosynthetic Genes Expressed in *Pseudomonas fluorescens* Strain P3 Improve the Induction of Systemic Resistance in Tobacco Against Tobacco Necrosis Virus. *Phytopathology*, **88**, 678-684.

93. Blumer, C., Heeb, S., Pessi, G. and Haas, D. (1999) Global GacA-steered control of cyanide and exoprotease production in *Pseudomonas fluorescens* involves specific ribosome binding sites. *Proc. Natl. Acad. Sci. USA*, **96**, 14073-14078.

94. Heeb, S., Blumer, C. and Haas, D. (2002) Regulatory RNA as mediator in GacA/RsmA-dependent global control of exoproduct formation in *Pseudomonas fluorescens* CHA0. *J. Bacteriol.*, **184**, 1046-1056.

Figure S1. RNA-Seq data processing workflow.

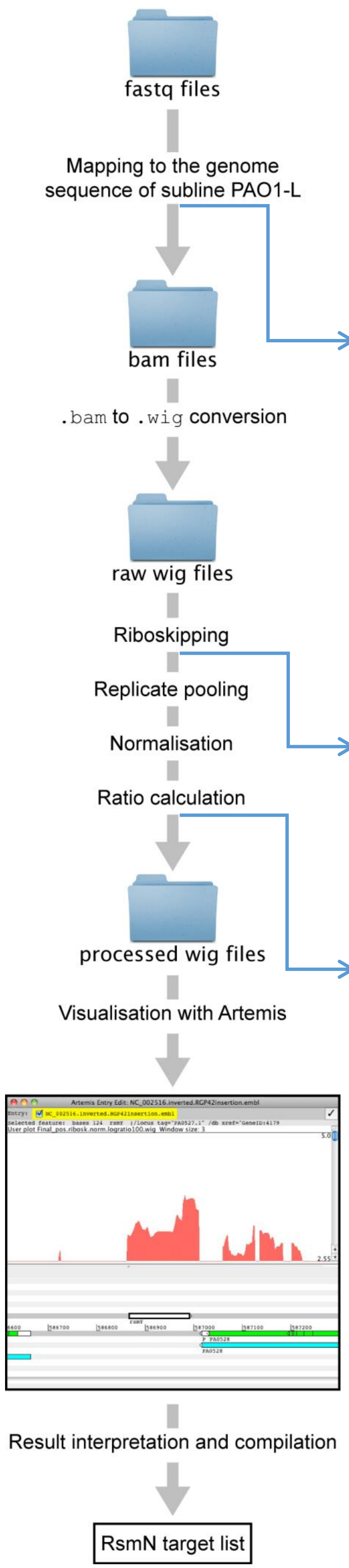

An approximation to the genome sequence of subline PAO1-L (not taking into account SNPs and INDELs) is obtained by reverting the *rrnA*/*rrnB* inversion in the reference sequence of subline PAO1-UW (GenBank NC_002516, reverse-complementing nucleotides 724'625-4'791'203) followed by inserting RGP42 (GenBank GQ141978) between nucleotides 5'242'103 and 5'242'104. This chromosomal arrangement is equivalent to that reported for subline PAO1-DSM (22).

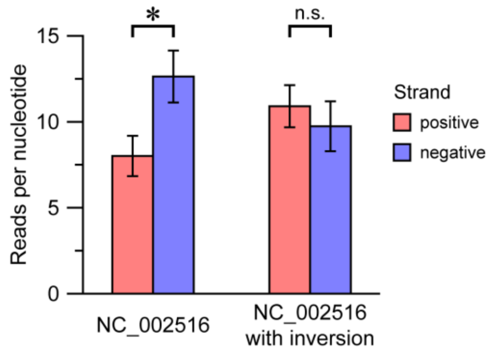

Mapping the reads to a genome arrangement that does not correspond to that of the subline used causes an apparent disequilibrium in the transcription levels of each strand. Correcting the *rrnA*/*rrnB* inversion in the reference sequence is therefore important for downstream strand-specific processing of the data, i.e. for normalisation.

Despite RNA sample ribodepletion and filtering during read mapping, strong signals from ribosomal operons were still present. Riboskipping consisted in zeroing all the values corresponding to these operons in the following regions:

Operon	begin	end
rrnA	721'775	727'262
rrnB	4'788'574	4'793'732
rrnC	5'276'152	5'281'666
rrnD	6'051'627	6'057'275

Normalisation is accomplished by dividing each wig value by the average reads per nucleotide in each corresponding file.

The enrichement ratio beween RsmN-bound RNA and total RNA is then calculated as follows:

$$ratio = \log\left(100 \times \frac{RNA_{RsmN-bound}}{RNA_{Total}}\right)$$

The cut-off value to consider potential RsmN targets was set to 2.55, which corresponds to an RNA 3.5-fold more abundant in the RsmN-bound samples than in the total RNA.

The result list is compiled manually following data visualisation and interpretation.

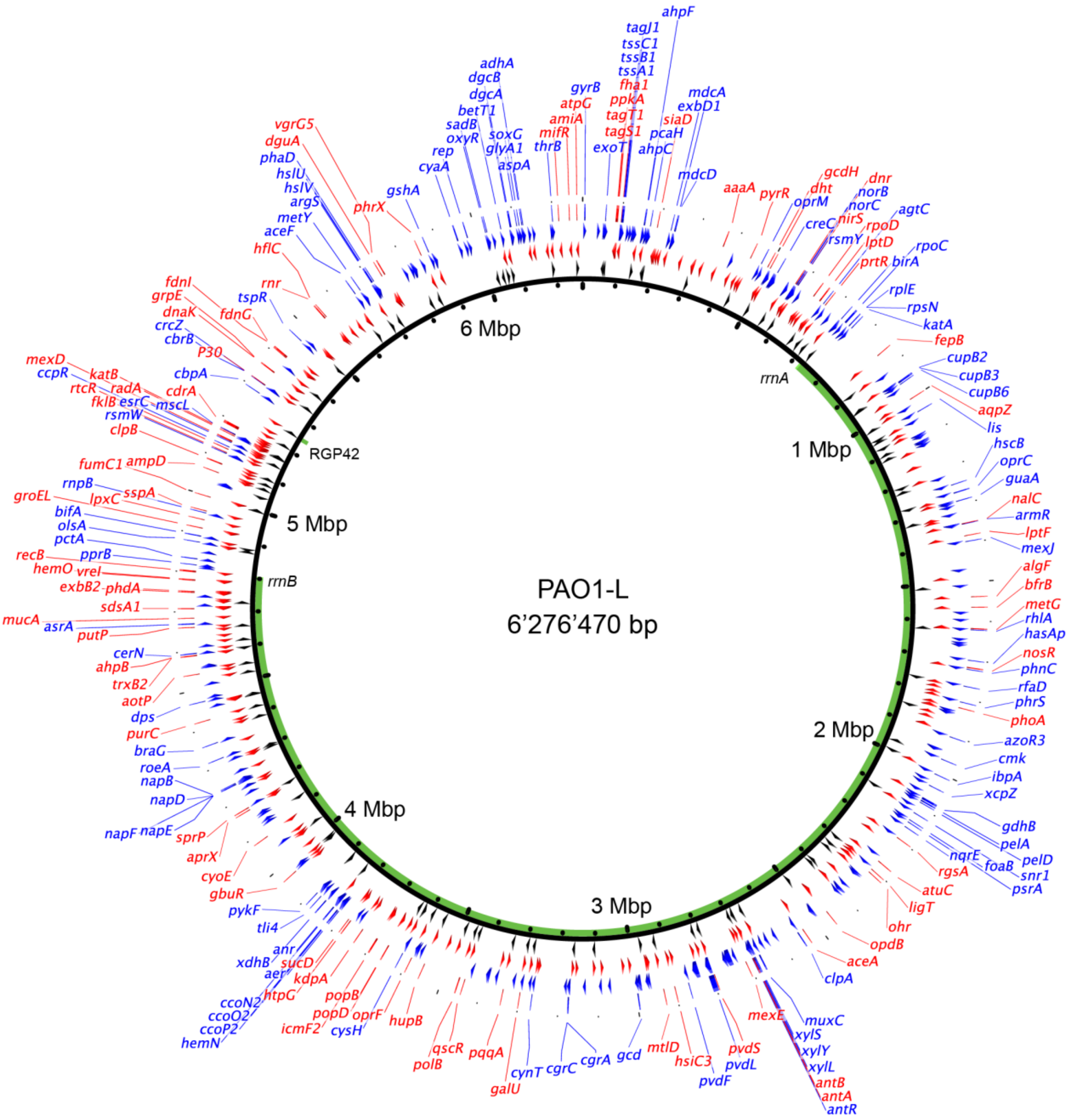

Figure S2. Genomic map of *P. aeruginosa* PAO1-L indicating the position of the 503 RsmN targets identified in this study. Transcripts were mapped to the chromosome of subline PAO1-L taking into account the known large *rrnA*/*rrnB* inversion (21, green arc) and the inclusion of RGP42 (22). Blue and red arrowheads indicate the positions and strand-specific orientations of the genes nearest to the RsmN-bound transcripts, and black arrowheads indicate transcripts that are not transcribed in the same direction as their nearest open reading frames or non-coding RNAs. For clarity, only genes with assigned names have been labelled (for the complete list, see Supplementary Table S2).

Table S2. RsmN targets identified in this study. Sequenced transcripts bound to RsmN were mapped to the PAO1-L chromosome and genes to which they belonged were identified. Genes highlighted in grey indicate transcripts which positions and orientations are opposed to that of the nearest annotated genes, i.e. divergently transcribed (highlighted in purple) or transcribed in antisense (highlighted in red). The column 'RsmA control' indicates genes which RNA levels have been reported to be affected by an *rsmA* deletion (3), many of which can be expected to be direct RsmA targets.

Locus Tag	Gene	Product or function	RsmA control
PA0004	*gyrB*	DNA gyrase subunit B	
PA0040		conserved hypothetical protein	
PA0042		hypothetical protein	
PA0044	*exoT*	exoenzyme T	+
PA0046		hypothetical protein	
PA0072	*tagS1*	TagS1	
PA0073	*tagT1*	TagT1	
PA0074	*ppkA*	serine/threonine protein kinase PpkA	+
PA0081	*fha1*	Fha1	
PA0082	*tssA1*	TssA1	+
PA0083	*tssB1*	TssB1	+
PA0084	*tssC1*	TssC1	+
PA0086	*tagJ1*	TagJ1	
PA0097		hypothetical protein	
PA0103		probable sulfate transporter	
PA0111		hypothetical protein	
PA0113		probable cytochrome c oxidase assembly factor	
PA0118		hypothetical protein	
PA0123		probable transcriptional regulator	
PA0139	*ahpC*	alkyl hydroperoxide reductase subunit C	
PA0140	*ahpF*	alkyl hydroperoxide reductase subunit F	
PA0141		conserved hypothetical protein	
PA0146		conserved hypothetical protein	
PA0147		probable oxidoreductase	
PA0151		probable TonB-dependent receptor	
PA0153	*pcaH*	protocatechuate 3,4-dioxygenase, beta subunit	
PA0169	*siaD*	SiaD	
PA0170		hypothetical protein	
PA0173		probable methylesterase	
PA0179		probable two-component response regulator	
PA0185		probable permease of ABC transporter	
PA0199	*exbD1*	transport protein ExbD	
PA0200		hypothetical protein	
PA0208	*mdcA*	malonate decarboxylase alpha subunit	+
PA0209		conserved hypothetical protein	+
PA0211	*mdcD*	malonate decarboxylase beta subunit	+
PA0241		probable major facilitator superfamily (MFS) transporter	+
PA0273		probable major facilitator superfamily (MFS) transporter	
PA0275		probable transcriptional regulator	
PA0295		probable periplasmic polyamine binding protein	+
PA0328	*aaaA*	arginine-specific autotransporter AaaA	+
PA0359		hypothetical protein	

PA0366		probable aldehyde dehydrogenase	
PA0387		conserved hypothetical protein	
PA0397		probable cation efflux system protein	
PA0403	*pyrR*	transcriptional regulator PyrR	
PA0427	*oprM*	Major intrinsic multiple antibiotic resistance efflux outer membrane protein OprM precursor	
PA0434		hypothetical protein	
PA0441	*dht*	dihydropyrimidinase	
PA0442		hypothetical protein	
PA0447	*gcdH*	glutaryl-CoA dehydrogenase	
PA0449		hypothetical protein	+
PA0451		conserved hypothetical protein	
PA0460		hypothetical protein	
PA0462		hypothetical protein	
PA0464	*creC*	two-component sensor CreC	
PA0466		hypothetical protein	
PA0497		hypothetical protein	
PA0505		hypothetical protein	
PA0506		probable acyl-CoA dehydrogenase	
PA0519	*nirS*	nitrite reductase precursor	
PA0521		probable cytochrome c oxidase subunit	
PA0523	*norC*	nitric-oxide reductase subunit C	
PA0524	*norB*	nitric-oxide reductase subunit B	
PA0525		probable dinitrification protein NorD	
PA0526		hypothetical protein	
PA0527	*dnr*	transcriptional regulator Dnr	
PA0527.1	*rsmY*	regulatory RNA RsmY	
PA0528		probable transcriptional regulator	
PA0545		hypothetical protein	
PA0563		conserved hypothetical protein	
PA0572		hypothetical protein	+
PA0576	*rpoD*	sigma factor RpoD	
PA0595	*lptD*	LPS assembly outer membrane protein LptD	
PA0599		hypothetical protein	
PA0605	*agtC*	probable permease of ABC transporter	
PA0611	*prtR*	transcriptional regulator PrtR	
PA0620		probable bacteriophage protein	
PA0643		hypothetical protein	
PA0659		hypothetical protein	+
PA0672	*hemO*	heme oxygenase	+
PA0675	*vreI*	ECF sigma factor, VreI	
PA0691	*phdA*	prevent-host-death protein A	
PA0693	*exbB2*	transport protein ExbB2	+
PA0695		hypothetical protein	
PA0702		hypothetical protein	
PA0714		hypothetical protein	
PA0718		hypothetical protein of bacteriophage Pf1	
PA0730		probable transferase	+
PA0737		hypothetical protein	

PA0738		conserved hypothetical protein	
PA0740	*sdsA1*	SDS hydrolase SdsA1	
PA0763	*mucA*	anti-sigma factor MucA	
PA0779	*asrA*	ATP-dependent Lon protease	
PA0783	*putP*	sodium/proline symporter PutP	
PA0789		probable amino acid permease	
PA0804		probable oxidoreductase	
PA0820		hypothetical protein	
PA0830		hypothetical protein	
PA0840		probable oxidoreductase	
PA0845	*cerN*	CerN	
PA0848	*ahpB*	Alkyl hydroperoxide reductase subunit AhpC (peroxiredoxin)	
PA0849	*trxB2*	thioredoxin reductase 2	
PA0875		conserved hypothetical protein	
PA0877		probable transcriptional regulator	
PA0884		probable C4-dicarboxylate-binding periplasmic protein	
PA0892	*aotP*	arginine/ornithine transport protein AotP	
PA0907		hypothetical protein	
PA0920		hypothetical protein	
PA0952		hypothetical protein	+
PA0962	*dps*	DNA-binding ferritin-like protein (oxidative damage protectant)	
PA0974		conserved hypothetical protein	
PA0975		probable radical activating enzyme	
PA0976		conserved hypothetical protein	
PA0980		hypothetical protein	
PA0982		hypothetical protein	
PA1013	*purC*	phosphoribosylaminoimidazole-succinocarboxamide synthase	
PA1031		conserved hypothetical protein	
PA1041		probable outer membrane protein precursor	
PA1070	*braG*	branched-chain amino acid transport protein BraG	
PA1090		hypothetical protein	
PA1107	*roeA*	RoeA	
PA1118		hypothetical protein	
PA1122		probable peptide deformylase	
PA1128		probable transcriptional regulator	
PA1153		hypothetical protein	
PA1157		probable two-component response regulator	+
PA1170		conserved hypothetical protein	
PA1173	*napB*	cytochrome c-type protein NapB precursor	
PA1175	*napD*	NapD protein of periplasmic nitrate reductase	
PA1176	*napF*	ferredoxin protein NapF	
PA1177	*napE*	periplasmic nitrate reductase protein NapE	
PA1188		hypothetical protein	
PA1190		conserved hypothetical protein	
PA1191		hypothetical protein	
PA1196		probable transcriptional regulator	
PA1209		hypothetical protein	
PA1239		hypothetical protein	

PA1242	*sprP*	serine protease, subtilisin family	
PA1245	*aprX*	AprX	+
PA1262		probable major facilitator superfamily (MFS) transporter	
PA1267		hypothetical protein	
PA1282		probable major facilitator superfamily (MFS) transporter	
PA1289		hypothetical protein	
PA1321	*cyoE*	cytochrome o ubiquinol oxidase protein CyoE	
PA1351		probable sigma-70 factor, ECF subfamily	
PA1352		conserved hypothetical protein	
PA1365		probable siderophore receptor	
PA1369		hypothetical protein	
PA1394		hypothetical protein	
PA1414		hypothetical protein	+
PA1415		hypothetical protein	
PA1419		probable transporter	
PA1422	*gbuR*	GbuR	
PA1429		probable cation-transporting P-type ATPase	
PA1498	*pykF*	pyruvate kinase I	
PA1500		probable oxidoreductase	
PA1501		conserved hypothetical protein	
PA1509	*tli4*	Tli4	
PA1522		hypothetical protein	
PA1523	*xdhB*	xanthine dehydrogenase	
PA1541		probable drug efflux transporter	
PA1542		hypothetical protein	
PA1544	*anr*	transcriptional regulator Anr	
PA1545		hypothetical protein	
PA1546	*hemN*	oxygen-independent coproporphyrinogen III oxidase	
PA1555	*ccoP2*	Cytochrome c oxidase, cbb3-type, CcoP subunit	
PA1556	*ccoO2*	Cytochrome c oxidase, cbb3-type, CcoO subunit	
PA1557	*ccoN2*	Cytochrome c oxidase, cbb3-type, CcoN subunit	
PA1561	*aer*	aerotaxis receptor Aer	
PA1589	*sucD*	succinyl-CoA synthetase alpha chain	
PA1596	*htpG*	heat shock protein HtpG	
PA1604		hypothetical protein	
PA1613		hypothetical protein	
PA1624		hypothetical protein	
PA1633	*kdpA*	potassium-transporting ATPase, A chain	
PA1669	*icmF2*	IcmF2	
PA1673		hypothetical protein	+
PA1708	*popB*	translocator protein PopB	+
PA1709	*popD*	Translocator outer membrane protein PopD precursor	+
PA1735		hypothetical protein	
PA1752		hypothetical protein	
PA1755		hypothetical protein	
PA1756	*cysH*	3'-phosphoadenosine-5'-phosphosulfate reductase	
PA1777	*oprF*	Major porin and structural outer membrane porin OprF precursor	
PA1779		assimilatory nitrate reductase	

PA1781.1		P11
PA1789		hypothetical protein
PA1804	*hupB*	DNA-binding protein HU
PA1841		hypothetical protein
PA1866		hypothetical protein
PA1886	*polB*	DNA polymerase II
PA1898	*qscR*	quorum-sensing control repressor
PA1909		hypothetical protein
PA1940		hypothetical protein
PA1942		hypothetical protein
PA1964		probable ATP-binding component of ABC transporter
PA1972		conserved hypothetical protein
PA1985	*pqqA*	pyrroloquinoline quinone biosynthesis protein A
PA2006		probable major facilitator superfamily (MFS) transporter
PA2023	*galU*	UTP--glucose-1-phosphate uridylyltransferase
PA2024		probable ring-cleaving dioxygenase
PA2042		probable transporter (membrane subunit)
PA2044		hypothetical protein
PA2053	*cynT*	carbonate dehydratase
PA2057		hypothetical protein
PA2061		probable ATP-binding component of ABC transporter
PA2119		alcohol dehydrogenase (Zn-dependent)
PA2126	*cgrC*	CupA gene regulator C
PA2127	*cgrA*	CupA gene regulator A
PA2135		probable transporter
PA2146		conserved hypothetical protein
PA2161		hypothetical protein
PA2190		conserved hypothetical protein
PA2205		hypothetical protein
PA2212		conserved hypothetical protein
PA2225		hypothetical protein
PA2261		probable 2-ketogluconate kinase
PA2280		conserved hypothetical protein
PA2290	*gcd*	glucose dehydrogenase
PA2294		probable ATP-binding component of ABC transporter
PA2296		hypothetical protein
PA2297		probable ferredoxin
PA2298		probable oxidoreductase
PA2309		hypothetical protein
PA2315		hypothetical protein
PA2317		probable oxidoreductase
PA2318		hypothetical protein
PA2321		gluconokinase
PA2342	*mtlD*	mannitol dehydrogenase
PA2366	*hsiC3*	predicted component of the type VI protein secretion system
PA2376		probable transcriptional regulator
PA2396	*pvdF*	pyoverdine synthetase F
PA2402		probable non-ribosomal peptide synthetase

PA2403		hypothetical protein	
PA2420		probable porin	
PA2424	*pvdL*	PvdL	
PA2426	*pvdS*	sigma factor PvdS	
PA2459		hypothetical protein	
PA2460		hypothetical protein	
PA2462		hypothetical protein	
PA2469		probable transcriptional regulator	
PA2493	*mexE*	Resistance-Nodulation-Cell Division (RND) multidrug efflux membrane fusion protein MexE precursor	
PA2500		probable major facilitator superfamily (MFS) transporter	
PA2511	*antR*	AntR	
PA2512	*antA*	anthranilate dioxygenase large subunit	
PA2513	*antB*	anthranilate dioxygenase small subunit	
PA2515	*xylL*	cis-1,2-dihydroxycyclohexa-3,4-diene carboxylate dehydrogenase	+
PA2517	*xylY*	toluate 1,2-dioxygenase beta subunit	
PA2519	*xylS*	transcriptional regulator XylS	
PA2526	*muxC*	Multidrug efflux pump subunit AcrB	
PA2531		probable aminotransferase	
PA2538		hypothetical protein	
PA2550		probable acyl-CoA dehydrogenase	
PA2571		probable two-component sensor	
PA2620	*clpA*	ATP-binding protease component ClpA	
PA2634	*aceA*	isocitrate lyase AceA	
PA2676		probable type II secretion system protein	
PA2678		probable permease of ABC-2 transporter	
PA2699		hypothetical protein	
PA2700	*opdB*	proline porin OpdB	
PA2747		hypothetical protein	
PA2753		hypothetical protein	
PA2754		conserved hypothetical protein	
PA2756		hypothetical protein	
PA2759		hypothetical protein	
PA2778		hypothetical protein	
PA2790		hypothetical protein	
PA2822		conserved hypothetical protein	
PA2826		probable glutathione peroxidase	
PA2835		probable major facilitator superfamily (MFS) transporter	
PA2848		probable transcriptional regulator	
PA2850	*ohr*	organic hydroperoxide resistance protein	
PA2861	*ligT*	2'-5' RNA ligase	
PA2864		conserved hypothetical protein	
PA2868		hypothetical protein	
PA2888	*atuC*	geranyl-CoA carboxylase, beta-subunit	
PA2895		hypothetical protein	
PA2939		probable aminopeptidase	
PA2958.1	*rgsA*	RgsA	
PA2959		conserved hypothetical protein	
PA2995	*nqrE*	Na+-translocating NADH:quinone oxidoreductase subunit Nqr5	

PA3006	*psrA*	transcriptional regulator PsrA	
PA3013	*foaB*	fatty-acid oxidation complex beta-subunit	
PA3032	*snr1*	cytochrome c Snr1	
PA3040		conserved hypothetical protein	
PA3046		conserved hypothetical protein	+
PA3061	*pelD*	PelD	
PA3064	*pelA*	PelA	+
PA3068	*gdhB*	NAD-dependent glutamate dehydrogenase	
PA3074		hypothetical protein	
PA3079		hypothetical protein	
PA3095	*xcpZ*	general secretion pathway protein M	
PA3106		probable short-chain dehydrogenase	
PA3126	*ibpA*	heat-shock protein IbpA	
PA3136		probable secretion protein	
PA3163	*cmk*	cytidylate kinase	
PA3179		conserved hypothetical protein	
PA3181		2-keto-3-deoxy-6-phosphogluconate aldolase	
PA3223	*azoR3*	AzoR3, azoreductase 3	
PA3237		hypothetical protein	
PA3238		hypothetical protein	
PA3261		hypothetical protein	
PA3271		probable two-component sensor	
PA3277		probable short-chain dehydrogenase	
PA3278		hypothetical protein	+
PA3283		conserved hypothetical protein	
PA3284		hypothetical protein	
PA3287		conserved hypothetical protein	
PA3289		hypothetical protein	
PA3296	*phoA*	alkaline phosphatase	+
PA3305.1	*phrS*	PhrS	
PA3309		conserved hypothetical protein	
PA3317		hypothetical protein	
PA3327		probable non-ribosomal peptide synthetase	
PA3336		probable major facilitator superfamily (MFS) transporter	
PA3337	*rfaD*	ADP-L-glycero-D-mannoheptose 6-epimerase	
PA3384	*phnC*	ATP-binding component of ABC phosphonate transporter	+
PA3391	*nosR*	regulatory protein NosR	
PA3407	*hasAp*	heme acquisition protein HasAp	+
PA3444		conserved hypothetical protein	
PA3450		probable antioxidant protein	
PA3458		probable transcriptional regulator	
PA3479	*rhlA*	rhamnosyltransferase chain A	
PA3482	*metG*	methionyl-tRNA synthetase	
PA3483		hypothetical protein	
PA3498		probable oxidoreductase	
PA3518		hypothetical protein	
PA3531	*bfrB*	bacterioferritin	
PA3550	*algF*	alginate o-acetyltransferase AlgF	

PA3573		probable major facilitator superfamily (MFS) transporter	
PA3594		probable transcriptional regulator	
PA3613		hypothetical protein	
PA3677	*mexJ*	multidrug efflux pump subunit AcrA	
PA3692	*lptF*	Lipotoxon F, LptF	
PA3718		probable major facilitator superfamily (MFS) transporter	
PA3719	*armR*	antirepressor for MexR, ArmR	
PA3720		hypothetical protein	
PA3721	*nalC*	NalC	
PA3734		hypothetical protein	
PA3739		probable sodium/hydrogen antiporter	
PA3741		hypothetical protein	
PA3762		hypothetical protein	+
PA3766		probable aromatic amino acid transporter	
PA3769	*guaA*	GMP synthase	
PA3772		hypothetical protein	
PA3781		probable transporter	
PA3790	*oprC*	Putative copper transport outer membrane porin OprC precursor	
PA3811	*hscB*	heat shock protein HscB	
PA3835		hypothetical protein	
PA3843		hypothetical protein	
PA3880		conserved hypothetical protein	
PA3881		hypothetical protein	+
PA3920		probable metal transporting P-type ATPase	
PA3921		probable transcriptional regulator	
PA3924		probable medium-chain acyl-CoA ligase	
PA3926		probable major facilitator superfamily (MFS) transporter	
PA3928		hypothetical protein	
PA3938		probable periplasmic taurine-binding protein precursor	
PA3943		conserved hypothetical protein	
PA3944		conserved hypothetical protein	
PA3954		hypothetical protein	
PA3967		hypothetical protein	
PA3979		hypothetical protein	
PA3983		conserved hypothetical protein	
PA3996	*lis*	lipoate synthase	
PA4019		probable aromatic acid decarboxylase	
PA4022		probable aldehyde dehydrogenase	
PA4034	*aqpZ*	aquaporin Z	
PA4061		probable thioredoxin	
PA4072		probable amino acid permease	
PA4081	*cupB6*	fimbrial subunit CupB6	
PA4084	*cupB3*	usher CupB3	
PA4085	*cupB2*	chaperone CupB2	
PA4095		hypothetical protein	+
PA4114		lysine decarboxylase	
PA4126		probable major facilitator superfamily (MFS) transporter	
PA4159	*fepB*	ferrienterobactin-binding periplasmic protein precursor FepB	

PA4236	*katA*	catalase	
PA4250	*rpsN*	30S ribosomal protein S14	
PA4251	*rplE*	50S ribosomal protein L5	
PA4269	*rpoC*	DNA-directed RNA polymerase beta* chain	
PA4277.3		tRNA-Tyr	
PA4280	*birA*	BirA bifunctional protein	
PA4284	*recB*	exodeoxyribonuclease V beta chain	
PA4292		probable phosphate transporter	
PA4296	*pprB*	two-component response regulator, PprB	
PA4309	*pctA*	chemotactic transducer PctA	+
PA4328		hypothetical protein	
PA4346		hypothetical protein	
PA4347		hypothetical protein	
PA4348		conserved hypothetical protein	
PA4351	*olsA*	1-acyl-sn-glycerol-3-phosphate acyltransferase	
PA4359		conserved hypothetical protein	
PA4367	*bifA*	BifA	
PA4385	*groEL*	GroEL protein	
PA4387		conserved hypothetical protein	
PA4406	*lpxC*	UDP-3-O-acyl-N-acetylglucosamine deacetylase	
PA4421.1	*rnpB*	RNA component of RNaseP, RnpB	
PA4428	*sspA*	stringent starvation protein A	
PA4470	*fumC1*	fumarate hydratase	
PA4471		hypothetical protein	
PA4500		probable binding protein component of ABC transporter	
PA4502		probable binding protein component of ABC transporter	
PA4518		hypothetical protein	
PA4522	*ampD*	beta-lactamase expression regulator AmpD	
PA4523		hypothetical protein	+
PA4539		hypothetical protein	
PA4542	*clpB*	ClpB protein	
PA4548		probable D-amino acid oxidase	
PA4570	*rsmW*	RsmW	
PA4571		probable cytochrome c	+
PA4572	*fklB*	peptidyl-prolyl cis-trans isomerase FklB	
PA4581	*rtcR*	transcriptional regulator RtcR	
PA4587	*ccpR*	cytochrome c551 peroxidase precursor	
PA4591		hypothetical protein	
PA4595		probable ATP-binding component of ABC transporter	
PA4596	*esrC*	stress-regulated repressor of the mexCD-oprJ multidrug efflux operon	
PA4598	*mexD*	Resistance-Nodulation-Cell Division (RND) multidrug efflux transporter MexD	
PA4605		conserved hypothetical protein	
PA4607		hypothetical protein	
PA4609	*radA*	DNA repair protein RadA	
PA4610		hypothetical protein	
PA4611		hypothetical protein	
PA4613	*katB*	catalase	
PA4614	*mscL*	conductance mechanosensitive channel	

PA4619		probable c-type cytochrome	
PA4623		hypothetical protein	
PA4625	*cdrA*	cyclic diguanylate-regulated TPS partner A, CdrA	+
PA4638		hypothetical protein	
PA4654		probable major facilitator superfamily (MFS) transporter	
PA4673.12		RGP42	
PA4704	*cbpA*	cAMP-binding protein A	
PA4726.1	*P36*	ncRNA P36	
PA4726.11	*crcZ*	CrcZ	
PA4726.2	*P30*	P30	
PA4738		conserved hypothetical protein	
PA4761	*dnaK*	DnaK protein	
PA4762	*grpE*	heat shock protein GrpE	
PA4781		cyclic di-GMP phosphodiesterase	
PA4790		conserved hypothetical protein	
PA4810	*fdnI*	nitrate-inducible formate dehydrogenase, gamma subunit	
PA4812	*fdnG*	formate dehydrogenase-O, major subunit	
PA4822		hypothetical protein	+
PA4857	*tspR*	small neutral amino acid transporter SnatA, MarC family	
PA4869		hypothetical protein	
PA4879		conserved hypothetical protein	
PA4884		hypothetical protein	
PA4937	*rnr*	exoribonuclease RNase R	
PA4941	*hflC*	protease subunit HflC	
PA4963		hypothetical protein	
PA4982		probable two-component sensor	
PA5005		probable carbamoyl transferase	
PA5006		hypothetical protein	
PA5016	*aceF*	dihydrolipoamide acetyltransferase	
PA5025	*metY*	homocysteine synthase	
PA5027		hypothetical protein	
PA5037		hypothetical protein	
PA5051	*argS*	arginyl-tRNA synthetase	
PA5053	*hslV*	heat shock protein HslV	
PA5054	*hslU*	heat shock protein HslU	
PA5057	*phaD*	poly(3-hydroxyalkanoic acid) depolymerase	
PA5084	*dguA*	glycine/D-amino acid oxidase	
PA5086		hypothetical protein	
PA5090	*vgrG5*	Uncharacterized conserved protein, implicated in type VI secretion and phage assembly	
PA5132		hypothetical protein	
PA5149		conserved hypothetical protein	
PA5156		hypothetical protein	
PA5157		probable transcriptional regulator	
PA5158		probable outer membrane protein precursor	
PA5159		multidrug resistance protein	
PA5179		probable transcriptional regulator	
PA5183.01	*phrX*	ncRNA phrX	
PA5203	*gshA*	glutamate--cysteine ligase	

PA5207		probable phosphate transporter	
PA5210		probable secretion pathway ATPase	
PA5211		conserved hypothetical protein	
PA5272	*cyaA*	adenylate cyclase	
PA5290		conserved hypothetical protein	+
PA5296	*rep*	ATP-dependent DNA helicase Rep	
PA5344	*oxyR*	OxyR	
PA5346	*sadB*	SadB	
PA5362		conserved hypothetical protein	
PA5370		probable major facilitator superfamily (MFS) transporter	
PA5375	*betT1*	choline transporter BetT	
PA5381		hypothetical protein	
PA5398	*dgcA*	DgcA, Dimethylglycine catabolism	+
PA5399	*dgcB*	DgcB, Dimethylglycine catabolism	
PA5415	*glyA1*	serine hydroxymethyltransferase	+
PA5419	*soxG*	sarcosine oxidase gamma subunit	+
PA5427	*adhA*	alcohol dehydrogenase	
PA5429	*aspA*	aspartate ammonia-lyase	
PA5435		probable transcarboxylase subunit	+
PA5440		probable peptidase	
PA5442		conserved hypothetical protein	
PA5446		hypothetical protein	
PA5475		hypothetical protein	
PA5487		hypothetical protein	
PA5492		conserved hypothetical protein	
PA5495	*thrB*	homoserine kinase	
PA5511	*mifR*	MifR	
PA5538	*amiA*	N-acetylmuramoyl-L-alanine amidase	
PA5555	*atpG*	ATP synthase gamma chain	

Figure S3. To verify post-transcriptional control by RsmN and RsmA on *fhA1* expression, a third translational reporter was constructed by substituting the *fhA1* native SD by the non-RsmA-regulated SD of *lacZ* (*fhA1*-SD$_{lacZ}$'-'*luxCDABE*). Values given are averages from three different cultures ± standard deviation and correspond to the area under the curve (AUC) derived from plotting relative light units normalized to culture density (RLU/OD600) over time (24 h), and as percentage of the corresponding activity obtained in the WT (set at 100%).

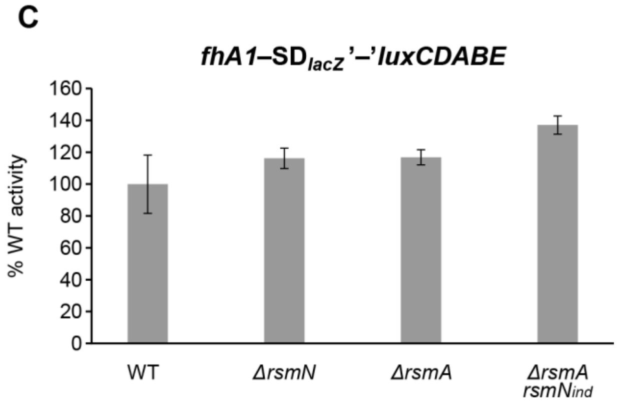

Figure S4.
Supernatant protein profiling from an equivalent number of *P. aeruginosa* cells from the strains indicated. Over-expression of *rsmN* in the *rsmAN* double mutant significantly reduced the abundance of a ~17 kDa band (red arrow), corresponding to the T6SS effector Hcp1 (96% sequencing coverage).

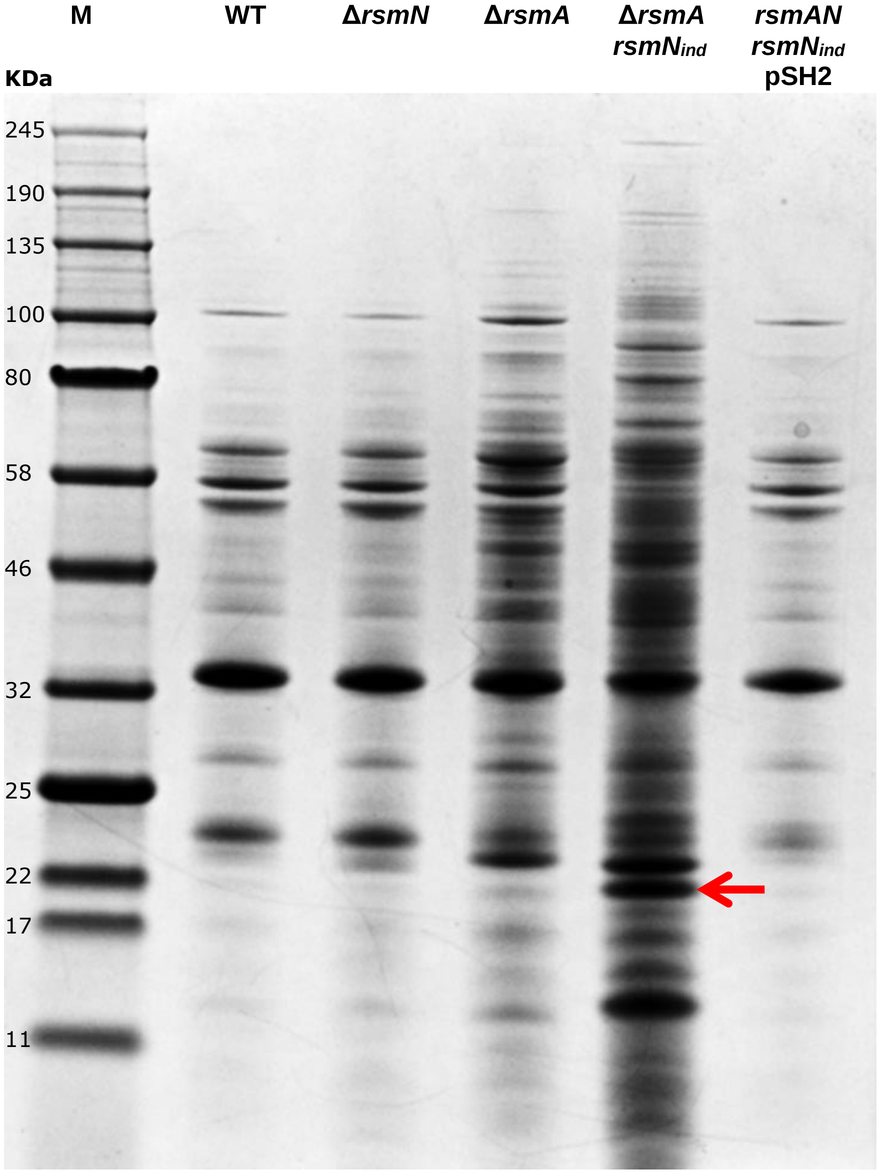

Table S3. Survival of *E. coli* DH5α (prey) was determined by measuring colony forming units (CFU) following 5 h exposure to the *P. aeruginosa* strains. The data represent the averages ± standard deviations from three independent experiments.

**Predator strain**	**CFUx10^{6}**
none	296.67±30.55
PAO1 / pME6000	39.67±11.5
Δ*rsmN* / pME6000	21.34±4.93
Δ*rsmA* / pME6000	5.26±1.58
Δ*rsmArsmN*$_{ind}$ / pME6000	0.16±0.06
Δ*rsmArsmN*$_{ind}$ / pHS2	39.34±2.31

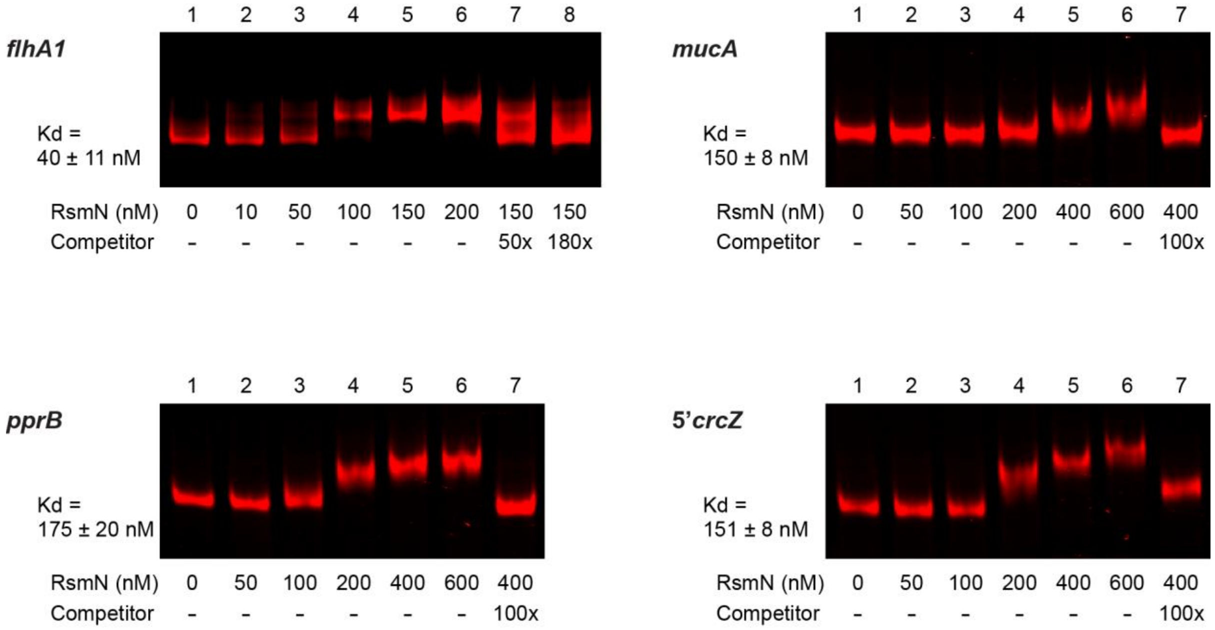

Figure S5. Repetition of the RsmN EMSAs appearing in Figs. 2A, 3A, 5A and 6A. Image analysis and the apparent dissociation constants (Kd averages ± standard deviations) obtained for each EMSA experiment were estimated three times using Image Studio V5.0 and GraphPad Prism V7 software. Non-labelled RsmY was used as specific competitor RNA at the excess concentrations as indicated in the Materials and Methods.

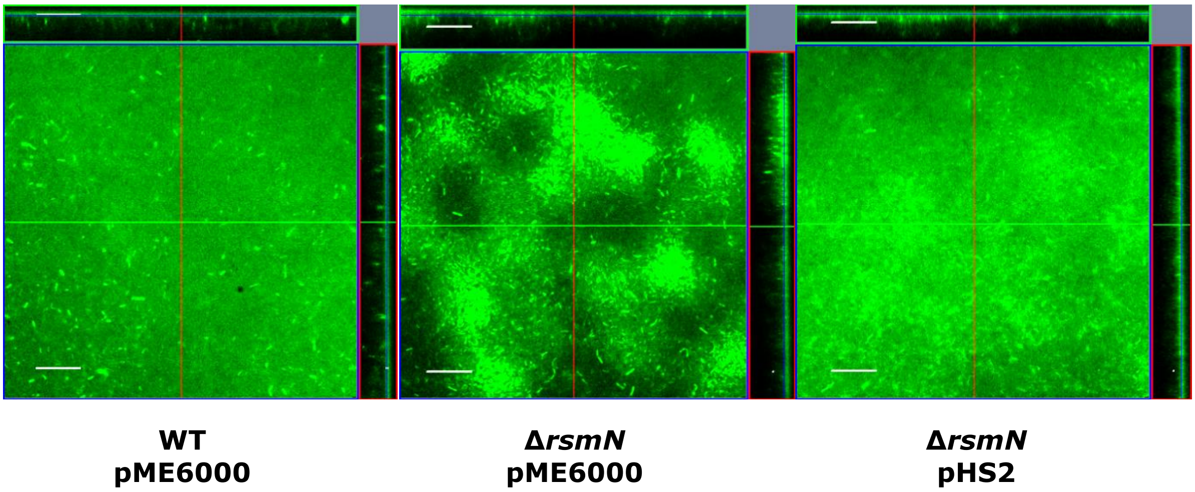

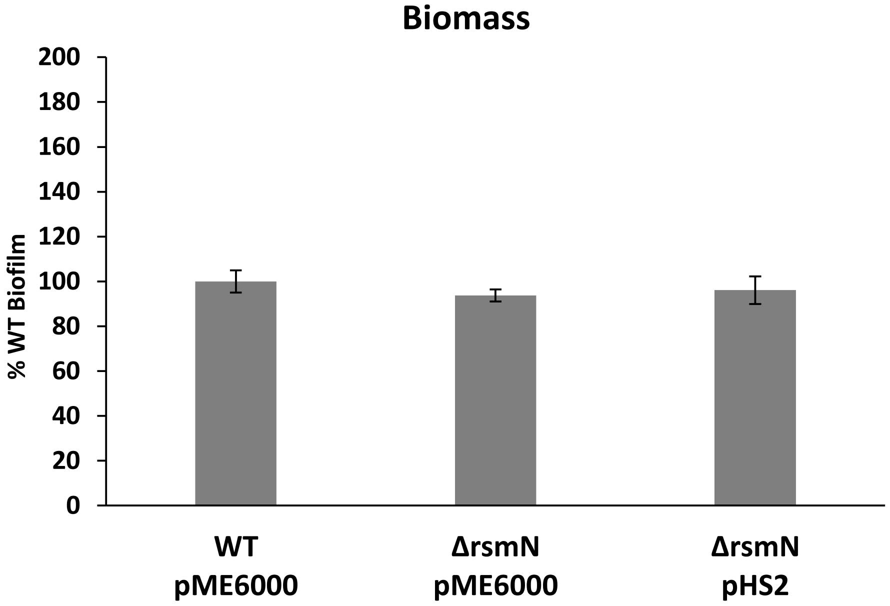

Figure S6. Influence on biofilm formation by RsmN in *P. aeruginosa* PAO1-N. (A) Confocal microscopy images of biofilms grown for 14 h in microfluidic chambers. Scale bars correspond to 20 µm. Bacteria were incubated at 37°C and stained with Syto-9 fluorescent dye for visualization and quantification. (B) Biofilm biomass was quantified using Comstat2 from four image stacks of biofilms formed by the different strains. Values reported are normalized as percentage of WT (set at 100%).

A

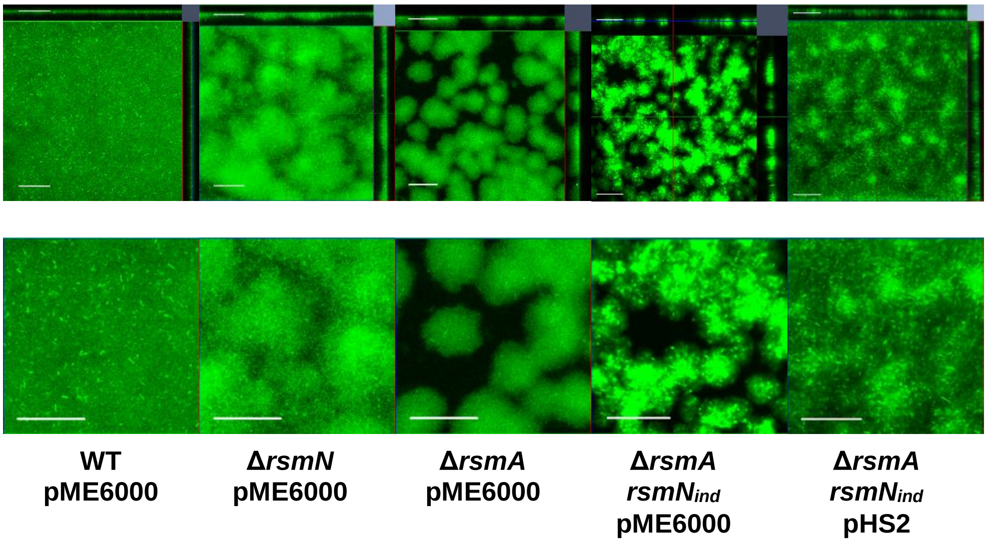

B

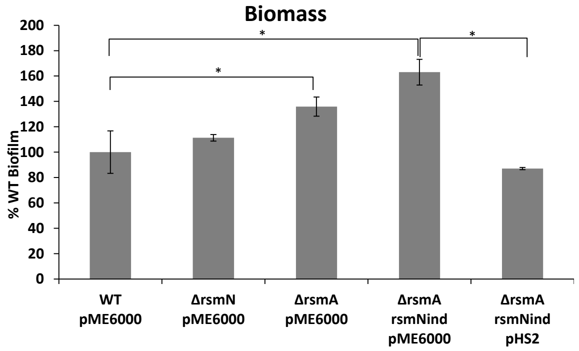

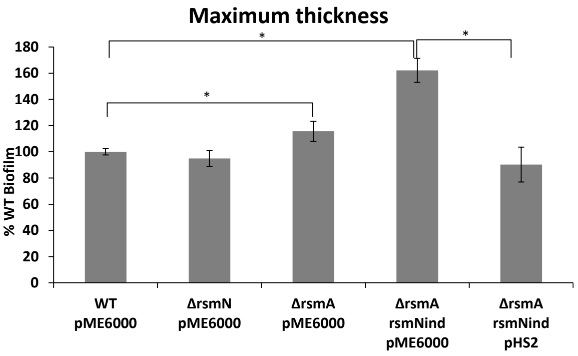

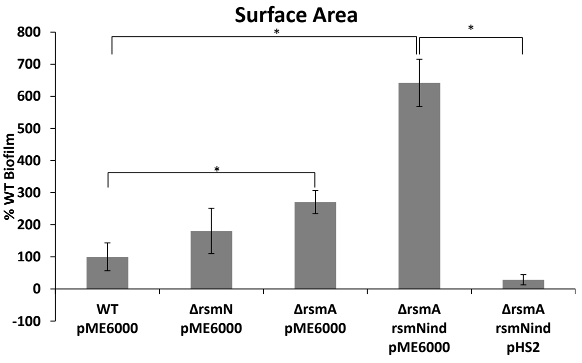

Figure S7. Control of biofilm formation by the Rsm system in *P. aeruginosa* PAO1-N WT and isogenic *rsm* mutants. (A) Confocal microscopy images of biofilms grown for 14 h in microfluidic chambers. Scale bars correspond to 50 µm. Bacteria were incubated at 37°C and stained with Syto-9 fluorescent dye for visualization and quantification. Lower row pictures are 4× magnifications of the upper-left quadrants of each corresponding picture in the upper row. (B) Biofilm biomass, maximum thickness and surface area were quantified using Comstat2 from four image stacks of biofilms formed by the different strains. Values reported are normalized as percentage of WT (set at 100%). Statistical differences were determined using a one-way ANOVA test (*$p<0.05$).

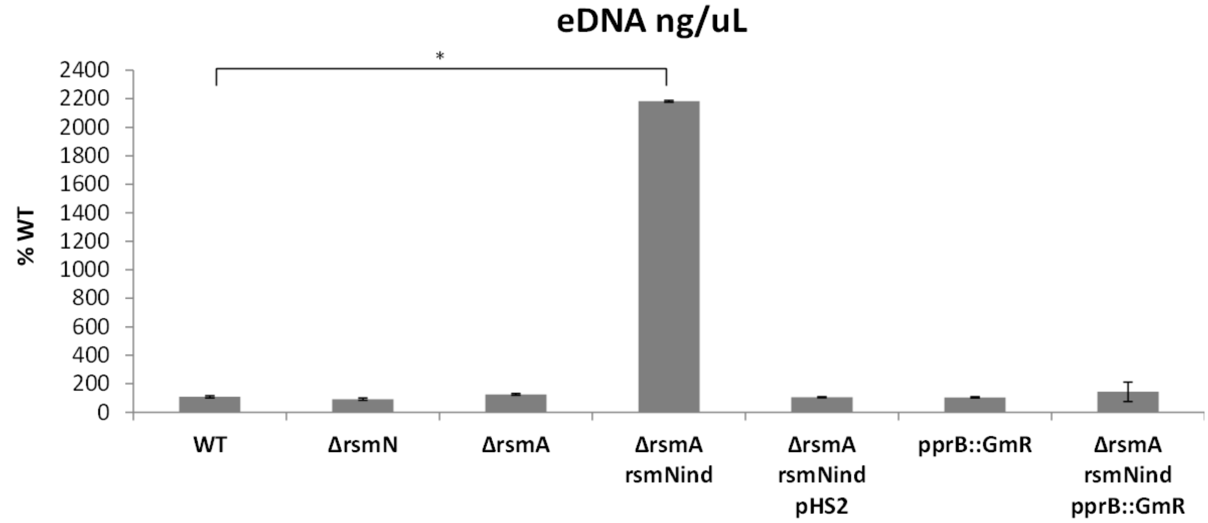

Figure S8. Control of extracellular DNA (eDNA) release by the Rsm system in planktonic cultures of *P. aeruginosa* PAO1-N WT and *rsm* mutants. eDNA from filtered supernatants was precipitated and the concentration determined using a NanoDrop spectrophotometer. Measurements were carried out in triplicate for each strain and values reported are normalized to percent of WT quantities (set at 100%). Statistical differences were determined using a one-way ANOVA test (*p<0.05).

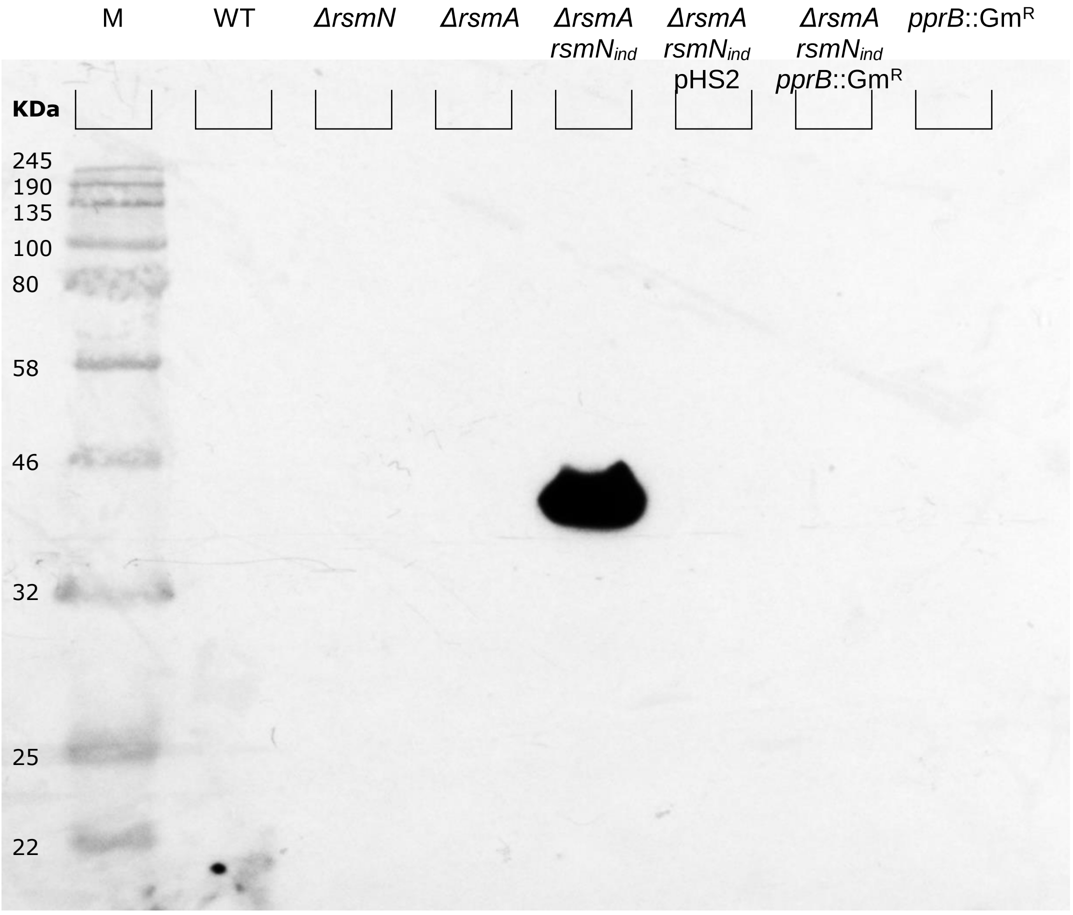

Figure S9. Control of autolysis by the Rsm system in planktonic cultures of *P. aeruginosa* PAO1-N WT and *rsm* mutants. Culture supernatants of strains indicated were harvested from an equivalent number of cells and the proteins immunoblotted for the cytoplasmic sigma factor RpoS to assess cell lysis.

Table S4. Characteristics and numerical data of the RNAs used for the EMSAs with RsmN.

		**First experiment**[1]		**Second experiment**[1]		**EMSA**
**RNA**	**Length** [1]	**Sum**	**Average**	**Sum**	**Average**	**Kd** (nM) [2]
fhA1	288	961	3.34	907	3.15	61 ± 26
5'*crcZ*	167	573	3.43	501	3.00	112 ± 43
mucA	244	547	2.24	614	2.52	148 ± 6
pprB	118	271	2.30	296	2.51	227 ± 60
pslA	151	291	1.93	243	1.61	>1000

[1]Genetic contexts and data (enrichment ratios) obtained are visualised in Supplementary Fig. S10.
[2]Observed Kd are averages ± standard deviations from the EMSA experiments (Figs. 2A, 3A, 5A, 6A and Supplementary Fig. S5).

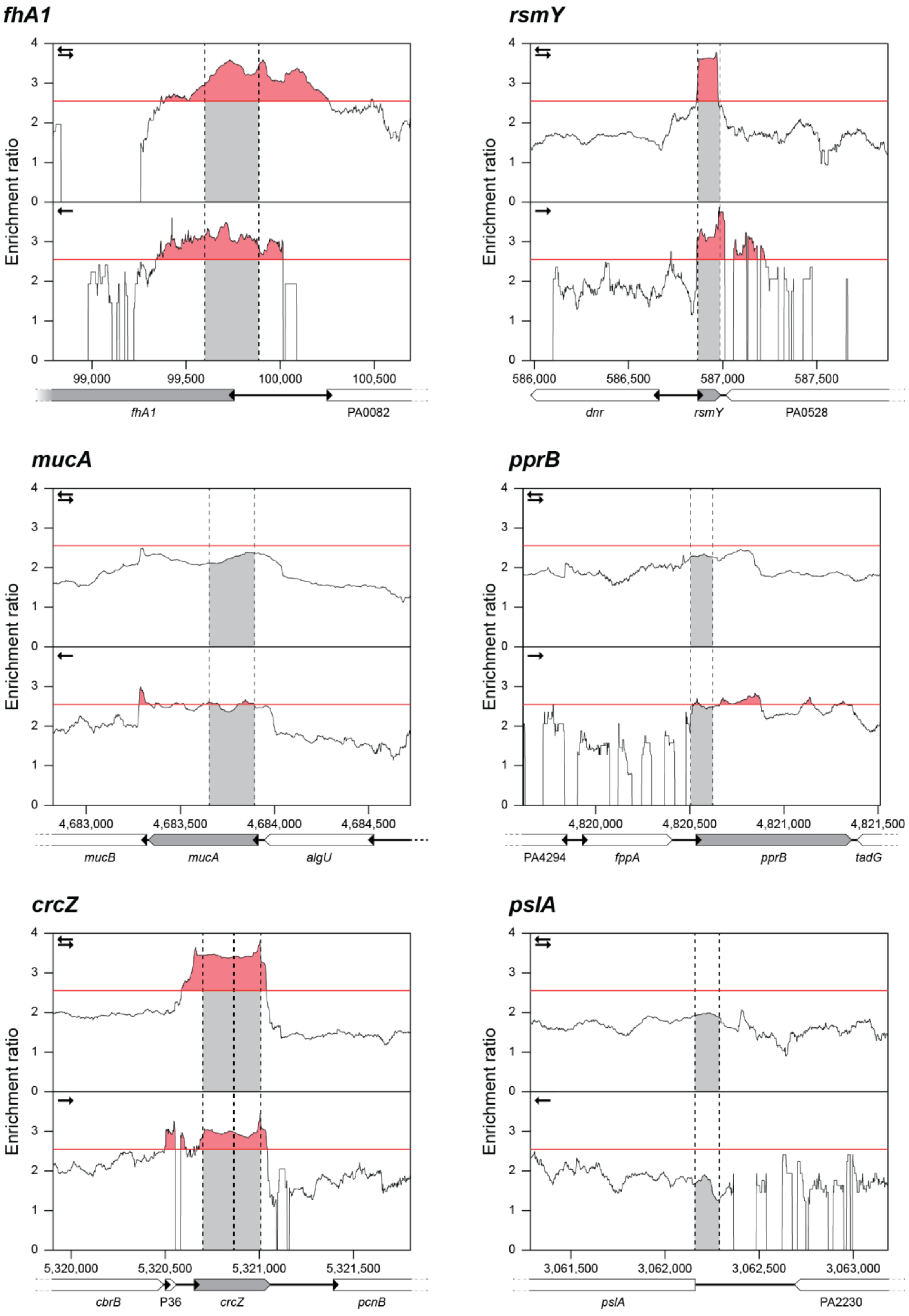

Figure S10. Genetic contexts and enrichment ratios of the regions used for the EMSAs. Ratios were plotted against their genomic positions and 1.9-kb snapshots centred on the regions transcribed *in vitro* for the EMSAs (shaded in grey) were taken. Values above the threshold (2.55, corresponding to a 3.5-fold enrichment, red line) are shaded in red. Strandless data from the first experiment are at the top (double arrows) and either positive or negative-strand data from the second experiment at the bottom (single arrows).
